# Getting off to a good start? Genetic evaluation of the *ex situ* conservation project of the Critically Endangered Montseny brook newt (*Calotriton arnoldi*)

**DOI:** 10.7717/peerj.3447

**Published:** 2017-06-13

**Authors:** Emilio Valbuena-Ureña, Anna Soler-Membrives, Sebastian Steinfartz, Mònica Alonso, Francesc Carbonell, Raquel Larios-Martín, Elena Obon, Salvador Carranza

**Affiliations:** 1 Unitat de Zoologia, Facultat de Biociències, Universitat Autònoma de Barcelona, Cerdanyola del Vallès, Barcelona, Catalonia, Spain; 2 Centre de Fauna Salvatge de Torreferrussa, Catalan Wildlife Service —Forestal Catalana, Santa Perpètua de la Mogoda, Barcelona, Catalonia, Spain; 3 Department of Evolutionary Biology, Zoological Institute, Technische Universität Braunschweig, Braunschweig, Germany; 4 Institute of Evolutionary Biology, CSIC-Universitat Pompeu Fabra, Barcelona, Catalonia, Spain

**Keywords:** *Ex situ*, Captive breeding program, Genetic monitoring, Genetic diversity, *Calotriton arnoldi*, Captive populations, Conservation, Critically endangered, Montseny brook newt, Microsatellite loci

## Abstract

*Ex situ* management strategies play an important role in the conservation of threatened species when the wild survival of the species cannot be ensured. Molecular markers have become an outstanding tool for the evaluation and management of captive breeding programs. Two main genetic objectives should be prioritized when planning breeding programs: the maintenance of maximum neutral genetic diversity, and to obtain “self-sustaining” captive populations. In this study, we use 24 microsatellite loci to analyze and evaluate the genetic representativity of the initial phases of the captive breeding program of the Montseny brook newt, *Calotriton arnoldi*, an Iberian endemic listed as Critically Endangered. The results show that the initial captive stock has 74–78% of the alleles present in the wild populations, and captures roughly 93–95% of their total genetic diversity as observed in a previous study on wild newts, although it does not reach the desired 97.5%. Moreover, the percentage of unrelatedness among individuals does not exceed 95%. Therefore, we conclude that the genetic diversity of the captive stock should be improved by incorporating genetic material from unrelated wild newts. In recognition of the previously described significant genetic and morphological differentiation between eastern and western wild populations of *C. arnoldi*, we suggest maintaining two distinct breeding lines, and we do not recommend outbreeding between these lines. Our comparisons of genetic diversity estimates between real and distinct sample-sized simulated populations corroborated that a minimum of 20 individuals are needed for each captive population, in order to match the level of genetic diversity present in the wild populations. Thus, the current initial stock should be reinforced by adding wild specimens. The captive stock and subsequent cohorts should be monitored in order to preserve genetic variation. In order to avoid genetic adaptation to captivity, occasionally incorporating previously genotyped individuals from the wild into the captive populations is recommended.

## Introduction

There is general agreement among scientists that we are in the midst of the sixth great mass extinction. Anthropogenic pressure is affecting natural environments both directly or indirectly (Wake & Vredenburg, 2008). Amphibians are amongst the most severely affected; being the vertebrate group catalogued globally at risk (Hoffmann et al., 2010), effective *in situ* conservation efforts, such as habitat and ecosystem protection or restoration, are urgent and should be prioritized. *Ex situ* measures, such as captive breeding, should be viewed as a last resort in species recovery (Snyder et al., 1996). Captive breeding programs are typically associated with many limitations: high economic costs, adaptation to captivity (Araki, Cooper & Blouin, 2007; Williams & Hoffman, 2009), poor success in reintroductions (Harding, Griffiths & Pavajeau, 2015; McCleery, Hostetler & Oli, 2014) and more (see below) have been reported. The need for such programs is justified only when it is essential for species’ survival, and should be implemented only after a careful evaluation of costs and benefits of all conservation alternatives (Dolman et al., 2015; McGowan, Traylor-Holzer & Leus, 2016). Often, primary threats such as habitat loss, disease, or overexploitation lead to small isolated populations, which in turn become highly susceptible to additional stochastic threats that can lead to population decline and, eventually, to extinction (often referred to as the ‘extinction vortex’; IUCN/SSC, 2014). Without excluding other *in situ* actions, captive breeding can play a crucial role in the recovery of some species for which effective alternatives are unavailable in the short term (Frankham, 1995; Griffiths & Pavajeau, 2008; Ralls & Ballou, 1986; Snyder et al., 1996; McGowan, Traylor-Holzer & Leus, 2016).

When a captive breeding program is recommended, the number of initial founders and the correct selection of individuals to maximize genetic diversity are key factors, as they will determine the genetic diversity present in the captive population (Frankham, Ballou & Briscoe, 2010; Mace, Pemberton & Stanley, 1992; Senner, 1980; Willis & Willis, 2010). Captive populations can often only represent a small subset of a natural population due to spatial or financial limitations of the organizations that manage them (Willoughby et al., 2015). Moreover, captive populations are frequently established when the wild populations of the species are at risk or have already suffered significant reductions in population size (Conde et al., 2011; Lyles & May, 1987; Traylor-Holzer, 2011; Williams & Hoffman, 2009), which limits the number of indivduals that can be collected to establish captive programs. For these reasons, captive populations may represent only a small fraction of the genetic diversity of the wild population (Ballou & Lacy, 1995; Mace, 1986; Ralls & Ballou, 1992), limiting the genetic diversity of the *ex situ* founders. If this is the case, the captive populations may experience a loss of genetic diversity and the accumulation of mildly deleterious alleles, which could lead to inbreeding depression, thereby jeopardizing the long-term viability of the captive populations (Lacy, 1997; Willis, 1993). Another problem associated with *ex situ* breeding programs is genetic adaptation to the captive environment. Captive environments differ from wild ones, and therefore the genetic variants favoured in captivity differ somewhat from those favoured in natural environments. Crucially, the genetic variants selected for captive conditions may be overwhelmingly disadvantageous in the natural environment, reducing reproductive fitness of captive individuals when released into wild environments (Frankham, 2008). It is then imperative that a population’s genetic adaptation to captivity be considered when managing breeding stocks (Ballou & Lacy, 1995; Frankham, 1995; Frankham, 2008; Princée, 1995; Williams & Hoffman, 2009). Carefully planned breeding programs are therefore required to minimize these potential threats.

Two main aspects must be considered when establishing a breeding program. First, there is broad agreement among wildlife managers that the retention of the 97.5% of the genetic diversity from wild populations should be a sufficient goal for the long-term viability of the founder populations (Edwards et al., 2014). However, in some cases, these values cannot be achieved without compromising the wild populations (Lacy, 1994; Frankham, Ballou & Briscoe, 2010). Under these circumstances, a genetic evaluation and an efficient selection of the founder stock is necessary to ensure the conservation of the highest levels of genetic variability possible. Second, it is estimated that a “self-sustaining” *ex situ* population must keep a minimum of 90% of the founder’s population genetic variability for the complete duration of the *ex situ* program; equivalent to the time required for habitat recovery in nature (Ballou et al., 2006; Soulé et al., 1986). Therefore, the capture of the maximum genetic diversity from the wild populations and the long-term conservation of a relatively high level of this genetic diversity within the captive populations are the two main challenges for a successful *ex situ* breeding program.

Genetic diversity is measured in terms of allelic diversity and heterozygosity (Ballou & Foose, 1996; Ballou et al., 2010). Allelic diversity may represent the evolutionary potential of a population and is an important value to consider for a population’s long-term viability; it plays an important role in the adaptation to environmental change (Ballou et al., 2010; Briscoe et al., 1992; Frankham, 2003; Princée, 1995). Allelic diversity may be affected by population bottlenecks, such as those that can arise as a result of the establishment of breeding stocks (Amos & Balmford, 2001). Heterozygosity can be characterized either as the observed or expected (i.e., according to expectations of Hardy-Weinberg Equilibrium; HDW) heterozygosity. The former is defined by the proportion of genetic loci for which the average individual in a population is heterozygous. The latter is the probability that two homologous genes randomly drawn from the population are distinct alleles (i.e., the mean heterozygosity that would exist in a population if it were in Hardy-Weinberg equilibrium: Höglund, 2009; Lacy, 1994). One of the key questions to be addressed when establishing a breeding stock is “how many individuals are needed in order to obtain and maintain the required levels of genetic diversity?” (Hale, Burg & Steeves, 2012). Previous studies have addressed this question by calculating the number of captive individuals needed to adequately represent the genetic diversity within wild populations using rarefaction algorithms (Hale, Burg & Steeves, 2012; Kalinowski, 2005). Overall, the number of founders that start a breeding program should be a balance between the genetic diversity captured from the wild and its sustainability, the number of individuals to be extracted from the endangered wild population without compromising their populations, and the capacity of the program to maintain the number of breeders and descendants (McGowan, Traylor-Holzer & Leus, 2016).

**Figure 1:**
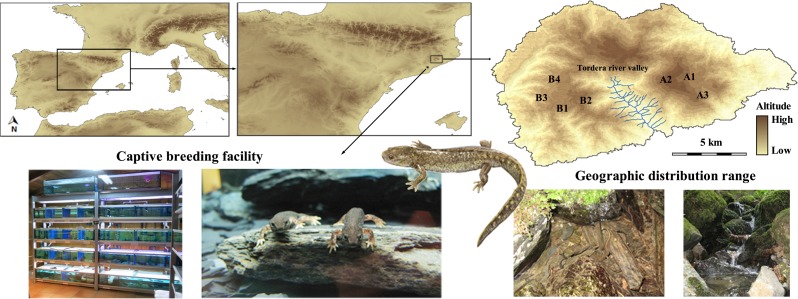
Location of the *Calotriton arnoldi* distribution range and the captive breeding facility. Localities of *C. arnoldi* represented do not correspond to the exact geographic locations intentionally due to conservation concerns. Source of the Montseny brook newt drawing: Toni Llobet.

The Montseny brook newt (*Calotriton arnoldi*) is one of the most threatened vertebrates in Europe, and is classified as Critically Endangered by the International Union for Conservation of Nature (IUCN) (Carranza & Martínez-Solano, 2009). Its current distribution is limited to an area of only 8 km^2^ in the Montseny Natural Park, NE Iberian Peninsula, Spain. Furthermore, although it is found in seven closely located brooks, its populations are divided into two main sectors along the Tordera river valley, which are separated by unsuitable habitat: the Eastern sector—three brooks (A1–A3); and the western sector—four brooks (B1–B4) (Fig. 1; Valbuena-Ureña, Amat & Carranza, 2013). With an estimated census population size of less than 1,500 mature individuals (Amat & Carranza, 2005), the major threats affecting this primarily water–dependent species are habitat degradation and disturbance, manifested as over-extraction of water for commercial purposes, deforestation, and the existence of tracks and roads that disrupt the continuity of the brooks where it occurs (Amat et al., 2014). Emergent diseases, such as *Batrachochytrium dendrobatidis* and *Batrachochytrium salamandrivorans*, have not yet affected this species, but should not be overlooked as they comprise an important threat to many amphibians, including newts and salamandrids (Martel et al., 2014; Obon et al., 2013; Spitzen-van der Sluijs et al., 2016).

Although the species conservation plan is not yet approved, some *in situ* management measures have been developed by the Barcelona Provincial Council, including habitat restoration and field monitoring (Amat et al., 2014). The IUCN guidelines recommend that captive breeding programs start when a species still numbers in the thousands. Considering the low number of individuals estimated for this species, the declining trend of its populations and the drying out of mountain streams, the need to start a captive breeding program immediately was fully justified. Thus, a provisional captive program was designed in 2007 by the Catalonian Government, in order to delve into the study and protection of this species. This work aims to maintain a genetic reserve that is representative of the wild populations, and to improve in its captive management while waiting for the results of detailed genetic studies (Catalan Government, 2010).

Following the recommendation of Amphibian Ark, and based on the precautionary principle, 20 individuals were captured in order to serve as founders for the captive population. As a result of preliminary data on the genetic and morphological variability between the two sectors (Carranza & Amat, 2005), founders from each sector were kept separately as two distinct breeding lines. Currently, a studbook for this species is being maintained using the Single Population Analysis and Record Keeping System (SPARKS), developed by the International Species Information System (ISIS) and the PMx program. However, it does not include genetic information about captive individuals. After ten years of operation, this program has successfully reared more than 1,700 individuals (Carbonell et al., 2016). Despite the viability of the breeding program, no analyses have yet been performed to determine the levels of genetic diversity among captive individuals, and to evaluate if the founders initially sampled are enough to satisfy the genetic goals of the captive breeding program.

The aim of this study is to use microsatellite loci to analyze the effectiveness of the current provisional breeding program of the Montseny brook newt (established with limited genetic information) and use this data to design the definitive captive stock in order to maximize its potential benefits to conservation. To do this, we evaluated whether the levels of genetic diversity (allelic diversity and heterozygosity) observed in the initial captive stock are comparable to the genetic diversity observed in the wild populations, and if this diversity is sufficient to be maintained over time. We also estimated the optimal numbers of individuals needed to ensure that the captive stocks accurately match the overall genetic diversity of the wild populations over time.

## Materials and Methods

### Sample collection and genotyping

Tissue samples were taken from 42 individuals (19 founders and 23 descendants) from the captive breeding facility (Table 1), and compared to results previously published from the wild populations (Valbuena-Ureña et al., 2017). Of the 19 founders, nine were from the eastern sector and belonged to population A1, and 10 were from the western sector and belonged to populations B1 and B2 (Fig. 1; see Valbuena-Ureña, Amat & Carranza (2013) for population codification). All F1 descendants were genotyped and are comprised of 17 F1 individuals from the eastern captive line, and six F1 individuals from the western captive line, all born in 2007. These descendants were interesting, as they originated from founder females that were already gravid when sampled from the wild. Therefore, they could exhibit genotypes from unsampled wild males. Due to spatial limitations in the facilities and readjustments of breeding methodology, only F1 experimental matings were designed; F2 is not yet available for genotyping. In total, 26 captive individuals from the eastern breeding line were analysed and compared to 77 genotyped wild animals from this sector (Valbuena-Ureña et al., 2017). Similarly, 16 captive individuals from the western breeding line were genotyped and compared to 83 wild animals from the western sector. The collection of all samples was conducted under the licenses required by the Catalan Government, with the permit numbers SF/298 and SF/469.

**Table 1:** Genetic diversities of the captive breeding lines compared to wild population sectors of the Montseny brook newt (*Calotriton arnoldi*). Values represent averages across 24 loci.

Group	*N*	*A*	*Ar*	*PA*	*H*_*O*_	*H*_*E*_	*F*_*IS*_
Eastern							
Founders	9	3.417	3.420	46	0.574	0.501	−0.088
F1	17	3.750	3.330	55	0.522	0.513	0.012
Total captive	26	4.083	3.380	57	0.540	0.519	−0.022
Wild*	77	4.167	4.112	75	0.544	0.538	**0.090**
Western							
Founders	10	2.917	2.650	28	0.413	0.421	0.072
F1	6	2.458	2.460	24	0.507	0.387	−0.227
Total captive	16	3.083	2.590	30	0.448	0.418	−0.039
Wild*	83	2.724	2.610	38	0.359	0.352	**0.184**

**Notes.**

*N* sample size *A* number of alleles per locus *Ar* allelic richness *PA* number of private alleles *H*_*O*_ observed heterozygosity *H*_*E*_ expected heterozygosity *F*_*IS*_ inbreeding coefficient

Values in bold indicate statistical significance after Bonferroni correction.

Wild* values were obtained from Valbuena-Ureña et al. (2017).

Tissue samples consisted of tail tips or fingers preserved in absolute ethanol. Genomic DNA was extracted using QiagenTM (Valencia, CA, USA) DNeasy Blood and Tissue Kit following the manufacturer’s protocol. For consistency, all 42 individuals were genotyped for the same 24 polymorphic microsatellite loci used to examine the wild populations (Drechsler et al., 2013; Valbuena-Ureña, Steinfartz & Carranza, 2014; Valbuena-Ureña et al., 2017). Details of the PCR-multiplex combinations of loci and specific amplification procedures are given in Valbuena-Ureña et al. (2017).

### Data analysis

MICRO-CHECKER (Van Oosterhout et al., 2004) was used to check for potential scoring errors, large allele dropout and the presence of null alleles. Pairwise linkage disequilibrium between loci and deviations from Hardy-Weinberg equilibrium in each grouping and for each locus were checked using the software GENEPOP version 4.2.1 (Rousset, 2008). Genetic diversity was measured for each grouping as the mean number of alleles (A), observed (*H*_*O*_), and expected heterozygosity (*H*_*E*_) using FSTAT 2.9.3.2 (Goudet, 1995). Formal analyses of raw allelic richness values were not performed, because the number of alleles detected at a locus can be influenced by sample sizes. Therefore a rarefied estimate of allelic richness (*Ar*) was obtained with HP-RARE (Kalinowski, 2005). The nonparametric Wilcoxon signed-rank test (Wilcoxon, 1945) was used to test for differences in diversity levels between captive and wild groups using locus-specific values of *A*, *Ar* and *H*_*E*_ as paired replicates. In analyses based on allelic richness, separate rarefactions were performed for each sector grouping, to account for the smallest sample size from a particular group. The observed number of private alleles (PA), defined here as alleles found in a single population throughout the study region, for each locus and each grouping was calculated with GDA (Lewis & Zaykin, 2000), and the ratio of presence compared to wild PA was computed. Further, overall and intrapopulation subdivision coefficients (*F*_*IS*_) for all markers and groupings were calculated using FSTAT 2.9.3.2, which calculates the estimator *f* of Weir & Cockerham (1984) for each marker, as well as a multilocus estimate. Genetic differentiation between the captive and the wild populations was derived from two parameters, *F*_*ST*_ and *R*_*ST*_ (Slatkin, 1995), values were obtained using ARLEQUIN 3.5.1.2 (Excoffier & Lischer, 2010).

### Relatedness among individuals, effective population size, and bottleneck detection

Pairwise values of genetic relatedness (*r*_*xy*_) were calculated using two estimators: the *r*_*qg*89_ estimator (Queller & Goodnight, 1989) and the *r*_*lr*99_ estimator (Lynch & Ritland , 1999). These estimated indices were compared following the Blouin et al. (1996) criteria implemented in *iREL* (Gonçalves da Silva & Russello, 2009), using the population allele frequencies estimated for each sample. The cut-off values were applied following the method described in Blouin et al. (1996), in order to classify individuals into relationship categories: unrelated (UR), half-siblings (HS), full siblings (FS) and parent–offspring (PO). Using this method, there is a greater than zero probability of misclassifying individuals if observed values of relatedness fall outside theoretically expected values (Russello & Amato, 2004). To minimize this error, the cut-off values specific for our samples were calculated using the Monte Carlo simulation procedure implemented in Blouin et al. (1996). In total, 10,000 dyads in each of the four relatedness categories were randomly generated using genotypes and allele frequencies specific for each eastern and western captive population as input data; *r*_*qg*89_ and *r*_*lr*99_ estimates for each simulated dyads were then computed. Expected misclassification rates were computed using the cut-off values described in Blouin et al. (1996) (the midpoints between the means of the distributions of pairwise relatedness estimates of each simulation category). Then, the index at which the overlap in the empirical distribution of unrelated and half-sibling dyads was minimized was selected. The relationship category compatible with the observed *r*_*xy*_ value was then determined for each individual pair. The observed distribution of pairwise relatedness among individuals for each population was plotted against those of the four simulated relatedness categories.

The effective population size (*N*_*e*_) for each breeding line based on one-sample *N*_*e*_ estimator method was calculated with ONeSAMP (Tallmon et al., 2008). ONeSAMP employs approximate Bayesian computation. The analyses were run specifying a minimum *N*_*e*_ value of 2 and a maximum of 100. To detect evidence of a recent bottleneck, we tested for significant heterozygosity excess or deficit with the program BOTTLENECK, version 1.2.02 (Piry, Luikart & Cornuet, 1999). This approach is effective for detecting bottlenecks that have occurred recently, within the past 0.2–4.0*N*_*e*_ generations (Luikart & Cornuet, 1998). The stepwise mutation (SMM) and two-phase mixed (TPM) models were tested with default parameters. We also sought signatures of genetic bottlenecks by calculating Garza and Williamson’s (Garza & Williamson, 2001) *M*-ratio for each breeding line using ARLEQUIN. The *M*-ratio is the mean ratio of the number of alleles to the range of allele size. When alleles are lost from a population, the number of alleles decreases at a faster rate than the allelic size range, so small (<0.68) *M*-ratio values are indicative of populations that have gone through a genetic bottleneck at some time in the past.

### Random subsampling of empirical datasets

In order to estimate how many captive individuals would be needed for each population to accurately match the level of the genetic diversity present in wild populations, comparisons of genetic diversity estimates between real and distinct sample-sized simulated populations were conducted following the methodology from Hale, Burg & Steeves (2012). A simulated dataset consisting of 100 replicates for each cluster was constructed, with the following sample sizes: 5, 10, 15, 20, 25, 30, 35, 40, 45 and 50 individuals. Each replicate contained a random subset of individuals from the empirical dataset, created using a macro in Excel. This was designed to assign a random number (between 1 and 10,000) to each individual from the empirical dataset, and then sort the dataset by this number. The macro selects the first five (or 10, 15, etc. depending on the sample size category) to a new worksheet, 100 times, resulting in 100 simulated ‘populations’ that are independent, i.e., random subsamples of the empirical dataset, at each sample size. Empirical datasets comprise all individuals genotyped, including wild and captive specimens. Sampling was done without replacement, so no individual was present more than once in the same replicate (as in a real population genetic dataset). As replicates were independent from each other, the same individual could be present in more than one replicate of the simulated dataset at each sample size. The mean expected heterozygosity (*H*_*E*_) and the mean percentage of alleles—including all alleles, common alleles (frequency >0.05) and rare alleles (frequency <0.05)—detected at each sample size (mean of the 100 random replicates per size) were then compared to each cluster (eastern sector, western sector, cluster A1A2 and cluster B1B2B4). The mean pairwise *F*_*ST*_ between the 100 random replicates at each sample size and the empirical dataset was also calculated for each dataset. In order to further examine variations between the simulated and empirical estimations, ANOVA with Tukey HSD post hoc tests were performed separately on data from each cluster using Statistica v.7.

## Results

A total of 140 alleles were identified in the 42 captive individuals analyzed, compared to the 170 alleles identified in the 160 individuals analyzed from the wild populations by Valbuena-Ureña et al. (2017). Regarding the presence of alleles by sector, 98 alleles were identified in the eastern captive breeding line. Ninety-five of these were present in the wild populations previously examined (making up 72% of alleles from the wild population); whereas three alleles were new. For the western captive breeding line, 74 alleles were found, of which one was new (not previously identified in the wild) and 73 were also present in the corresponding wild populations (78% of the wild alleles). Captive and wild allele frequencies are given in Table S1.

All 24 microsatellites were polymorphic for the eastern captive breeding line, but Us3 and Us7 were monomorphic throughout the western captive line (Table S2). Only Ca8 from the eastern sector was detected as a null allele. However, this does not suggest a locus-specific problem with null alleles, as no locus was affected by null alleles in all samples or in all populations. One locus (Calarn 52354) in the eastern captive line deviated from Hardy-Weinberg equilibrium (HWE), while the others did not show significant departures from HWE after applying Bonferroni correction (*p* > 0.00104). Linkage disequilibrium was only found between one pair of loci (Ca07 and Calarn52354 in the eastern captive group) after applying a Bonferroni correction (*p* > 0.00018).

Generally, higher allelic richness values were observed in both the eastern captive line and wild populations, in comparison to their western counterparts (Table 1). When comparing genetic diversity measures of captive individuals to wild ones (Valbuena-Ureña et al., 2017), similar values of mean number of alleles (A) are found in the eastern sector (*p* = 0.5135), while captive individuals from the western sector showed slightly higher values of A than wild specimens (*p* = 0.0012). Regarding differences among groups whilst taking into account sample size, rarefied allelic richness was lower in the captive individuals than in the wild ones. The difference was not statistically significant in the eastern sector (*p* = 0.0675), though it was in the western sector (*p* = 0.0007). These differences are almost certainly biologically significant, as it is impossible to retain genetic diversity that did not permeate into the captive population. Also, similar values of allelic richness were found among founders and descendants. The number of alleles per locus ranged from one to seven (Table S2). Each breeding line was found to contain private alleles (*PA*) in only one sector. A total of 57 PA were found in the eastern captive line (two of them not found in the wild populations) compared to 75 present in the wild populations (i.e., 76% PA representation in eastern captive line). In contrast, 81.5% PA representation was found in the western captive line (31of 38 PAs previously found in the wild). Regarding F1, 11 and two new PAs were found in the eastern and western captive breeding lines, respectively.

The mean expected heterozygosities (*H*_*E*_) were 0.519 and 0.418 in the eastern and western captive breeding lines, respectively. On a locus by locus basis, *H*_*E*_ of the captive eastern line was similar to the wild populations (*p* = 0.4750). No differences in *H*_*E*_ were observed between captive and wild individuals of the western populations (*p* = 0.8074), although a tendency towards smaller average heterozygosity was observed in the wild populations. Overall, *F*_*IS*_ did not show values significantly different from zero for each breeding line after applying Bonferroni correction. Pairwise *F*_*ST*_ and *R*_*ST*_ values revealed significant divergences among captive and wild counterparts. Values of *F*_*ST*_ and *R*_*ST*_ between the captive and wild populations was 0.061 and 0.036 ( *p* < 0.001) for the eastern sector, and 0.044 and 0.032 (*p* < 0.01) for the western sector.

### Relatedness among individuals, effective population size, and bottleneck detection

**Figure 2:**
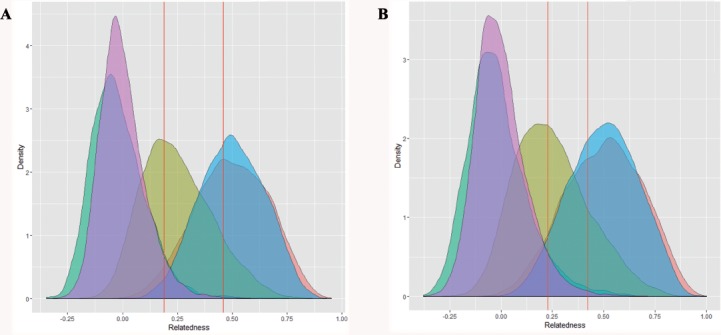
Observed distributions of relatedness (pink) for the (A) A1A2 dataset and (B) B1B2B4 dataset, plotted against expected distributions for 10,000 simulated pairs for each of the following relationship categories: nonrelated (green); half-siblings (yellow); full-siblings (red); parent–offspring (blue).

**Figure 3:**
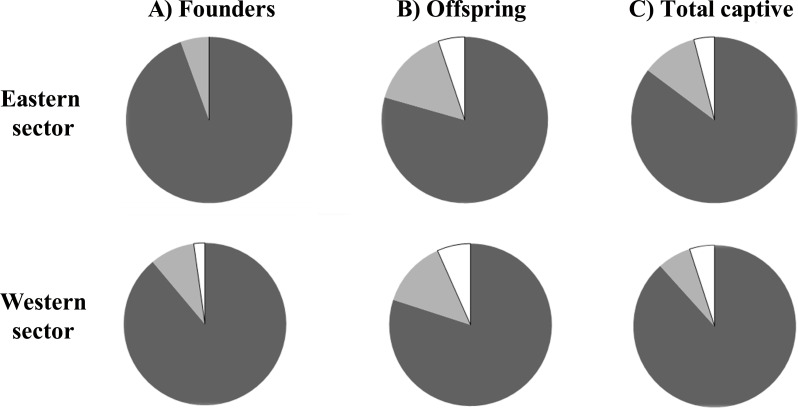
Proportion of individuals classified as unrelated (dark grey), first order relatives (full siblings and parent–offspring, white), and half-siblings (light grey) for each of the two *Calotriton arnoldi* breeding line (founders, offspring, total captive).

Comparison of the relatedness indices of *r*_*qg*89_ and *r*_*lr*99_ suggests that the latter performed better at discriminating between unrelated and half-sibling dyads, with fewer unrelated individuals being misclassified as half-sibling dyads following Blouin et al. (1996) (Table S3). All further analyses were therefore based upon the index of Lynch & Ritland (1999). The observed distribution of pairwise relatedness among individuals for each captive population was log normal, with a peak around zero (Fig. 2). Using the empirical distribution derived from 10,000 simulated dyad for each relatedness category, a dyad with a relatedness value of ≥0.1881 and 0.2295 for the eastern and western breeding line respectively, was determined to have a ≤ probability of 0.05 of being unrelated (Table S4). The proportion of dyads classified as unrelated for the eastern and western founder population is 94.44% and 88.89%, respectively (Fig. 3).

The estimated *N*_*e*_ for the eastern captive breeding line was 38.96 (median, 95% CI [34.27–52.25]), and *N*_*e*_ = 17.62 (median, 95% CI [15.30–24.05]) for the western captive breeding line (Table 2). Founders *N*_*e*_ estimates from both breeding lines were roughly 10, while effective population sizes estimated from the F1 were 17.03 and 7.97 for eastern and western captive founder stocks, respectively. No captive population showed any signs of a recent bottleneck under the TPM (one tailed test for heterozygote excess) or SMM models (Wilcoxon one-tailed test for excess of heterozygosity; *P* > 0.05 for all tests). In contrast, *M*-ratio tests suggest that both breeding lines experienced bottlenecks, with a Garza-Williamson *M*-ratio of 0.27 and 0.30 for the eastern and western breeding lines, substantially lower than the threshold of 0.68 considered indicative of a bottleneck.

**Table 2:** Estimates of effective population size (*N*_*e*_) for each breeding line calculated with ONeSAMP, with estimations of the upper and lower 95% CI.

Group	*N*_*e*_	95% Cis	
Eastern			
Founder	10.20	9.20	12.18
F1	17.03	15.07	22.12
Total captive	38.96	34.27	52.25
Western			
Founder	10.93	9.38	15.07
F1	7.97	6.93	10.22
Total captive	17.62	15.30	24.05

### Size of the captive lines needed to accurately represent the genetic diversity in wild populations

**Figure 4:**
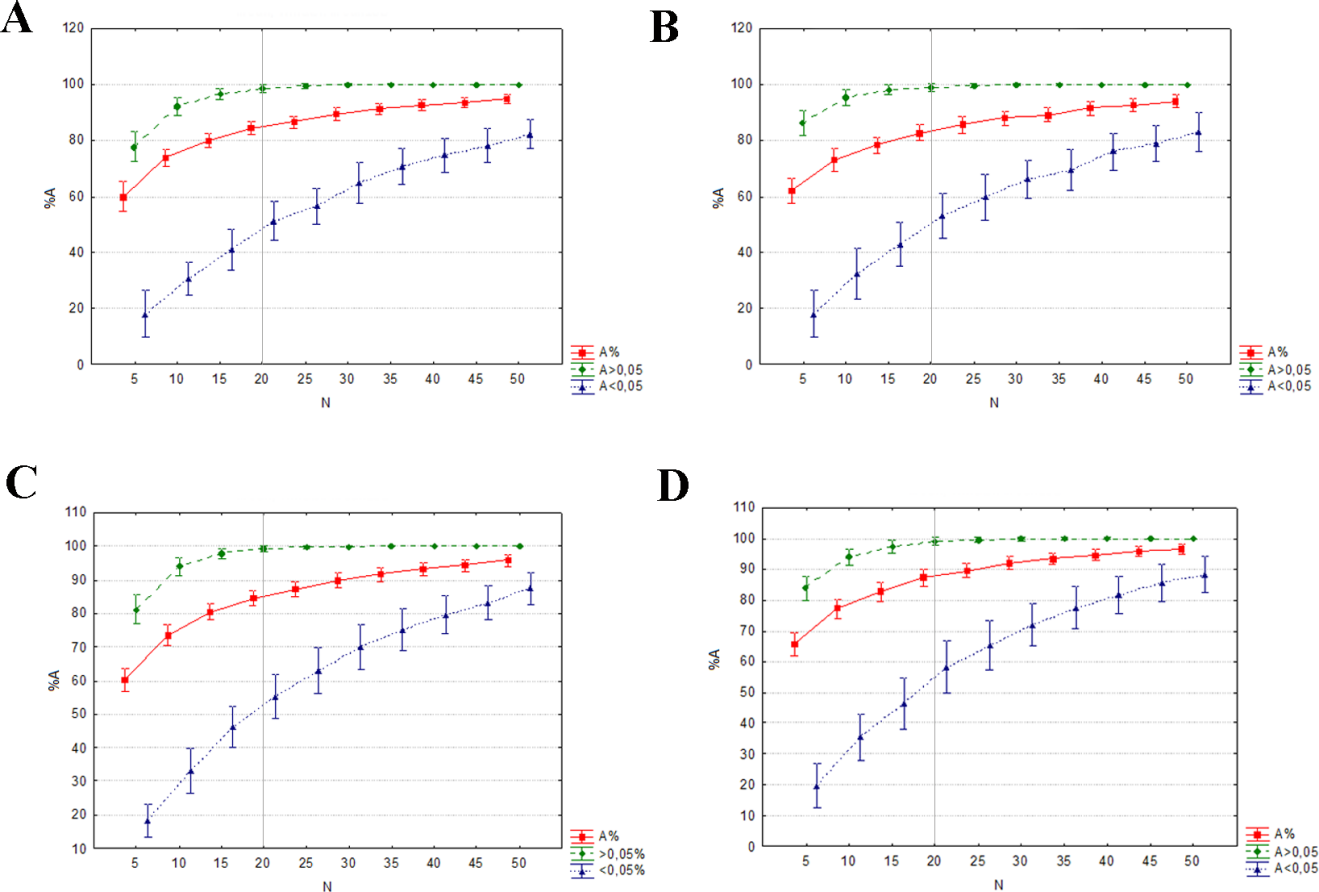
Mean percentage of all alleles (red), alleles at a frequency >0.05 (green) and alleles at frequency <0.05 (blue) detected at each sample size (of the 100 random replicates per size). The vertical grey lines show the sample size at which at least the 99% of the common alleles (frequency >0.05) were detected. (A) eastern sector; (B) western sector; (C) cluster A1A2; (D) cluster B1B2B4.

**Figure 5:**
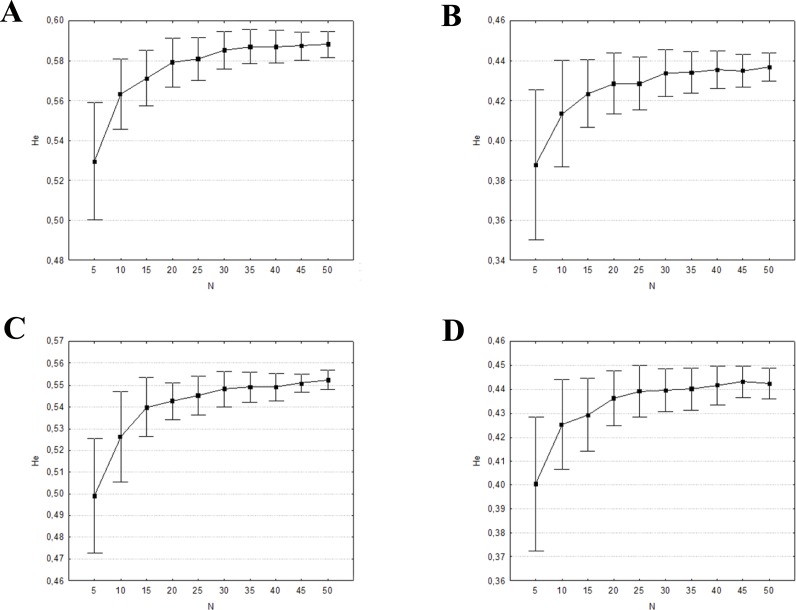
Mean and ± one standard deviation of expected heterozygosity (*H*_*E*_) for the 100 random replicates at each sample size. (A) eastern sector; (B) western sector; (C) cluster A1A2; (D) cluster B1B2B4.

**Figure 6:**
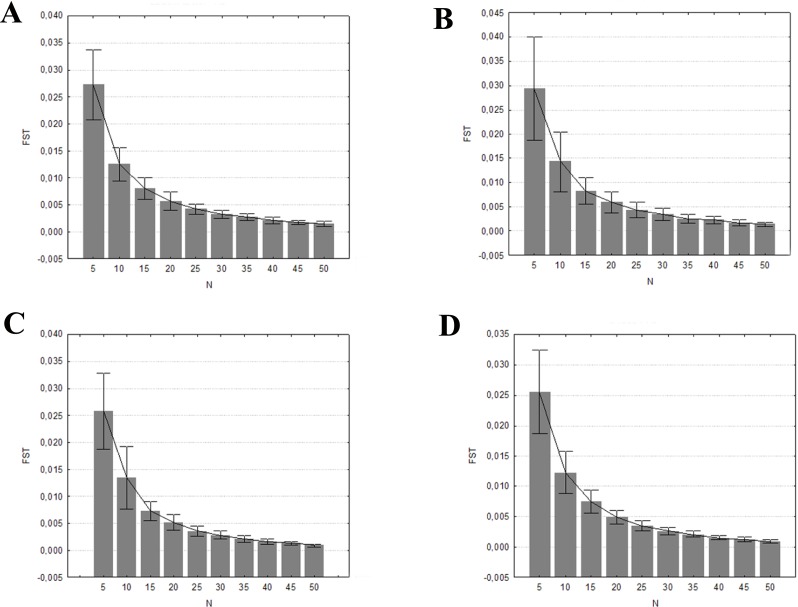
Mean pairwise *F*_*ST*_ and ± one standard deviation comparison between the 100 random replicates at each sample size and the empirical dataset. (A) Eastern sector; (B) western sector; (C) cluster A1A2; (D) cluster B1B2B4.

The mean percentages of allele detection were significantly different across the range of sample sizes (*P* < 0.001, in all four datasets) when considering all alleles, rare alleles, and common alleles (Table S5). In the two former cases, the Tukey HSD posthoc comparisons indicated significant differences among all sample size groups tested. The differences found when considering 20 individuals were not significant when only the common alleles were analysed. This is also the minimum number of individuals needed to detect at least the 99% of the common alleles (Fig. 4). The mean expected heterozygosities were far more precise (lower standard deviation) and accurate with increasing sampling size (ANOVA *P* < 0.001, in all four datasets), much of the increase occurring when the sample size grew from five to 20 individuals (Tukey HSD posthoc comparisons, Table S5; Fig. 5). Increasing sample size above 20 individuals seemed to have little impact on the mean *H*_*E*_ and its standard deviation for all four datasets. As above, the genetic distance between simulated and empirical datasets decreased as the sample size was increased, but again the decrease was not linear. ANOVAs indicated that Pairwise *F*_*ST*_ between each replicate and the empirical dataset were significantly different between sample sizes (*P* < 0.001, in all four datasets, Table S5). The difference was mainly from samples 5 to 20–25. No significant differences in pairwise *F*_*ST*_ was detected from 25 individuals upwards (Tukey HSD posthoc comparisons, Table S5), suggesting that there is little to be gained from sampling more than 25 individuals per captive population. In all four datasets, the 0.5% of genetic differentiation from wild populations is achieved with up to 20 individuals (Fig. 6), showing that the captive stock matches 99.5% of the wild genetic composition.

## Discussion

The main aims of the initial *ex situ* breeding program of *Calotriton arnoldi* when first established in 2007 were to maintain reservoir populations that reflected the genetic diversity found in the wild populations, to improve its captive management (produce and select the appropriate specimens for reintroduction), and to continue gathering knowledge on the biology of this species. The success of the program will depend largely on the genetic variability of the captive stock (Lacy, 1994). Decisions based on erroneous or incomplete genetic data may lead to a decrease of genetic diversity, jeopardizing the long-term survival of the captive stock and potentially negatively affecting the wild populations by the reintroduction of genetically depauperate captive specimens (Frankham, 1995; Hedrick & Kalinowski, 2000; Saccheri et al., 1998; Spielman, Brook & Frankham, 2004). This is the first study to evaluate the initial captive breeding program of the Critically Endangered Montseny brook newt. As such, these insights are crucial in ensuring the genetic quality of the breeding stock of the Montseny brook newt, and to successfully establish a long-term self-sustaining captive population. Furthermore, the methodology and conclusions reached herein can be used as an example for the genetic management of other *ex situ* conservation programs.

Here we discuss our two initial questions which we considered when establishing the *ex situ* program: (Q1) do we have a good genetic representation of neutral diversity from the wild populations? If not, (Q2) how large should the captive population be to reach this target? It is crucial to detect the point at which the genetic benefits gained by adding additional individuals to the captive populations is outweighed by the increased costs of sampling and maintaining those extra founders.

### Q1: estimation of the genetic diversity present in the captive stock

The levels of the genetic diversity measured in the captive dataset indicate that the individuals that form the current captive program are good, but not optimal, representatives of the genetic diversity present in the wild. Genetic theory indicates that the founder population should capture 97.5% of the genetic diversity present in the wild populations (Leus, Traylor-Holzer & Lacy, 2011). The current (initial) captive stock of *Calotriton arnoldi* captures roughly 93–95% of the total genetic diversity previously observed in the wild. Moreover, the percentage of unrelatedness among dyads from both breeding lines does not exceed 95%. Therefore, in order to increase genetic diversity, new genetic material should be incorporated by introducing new unrelated individuals or their sperm. Since this species present sperm storage (Alonso, 2013) and multiple paternity (Valbuena-Ureña et al., 2017), an ideal strategy to maximize the gene pool of the captive population while minimizing the impact on wild populations would be to incorporate wild-caught gravid females. Thus, descendants may retain the genetic diversity from individuals not kept in captivity.

Another issue to consider is that the genetic diversity of the captive stock should be maintained throughout the entire duration of the breeding program. A drop of the effective population size is detected on the eastern captive line (*N*_*e*_ = 39) when compared to the eastern wild populations (*N*_*e*_ = 86; Valbuena-Ureña et al., 2017), as well as in the western captive line (*N*_*e*_ = 18) compared to the western wild populations (*N*_*e*_ = 80). Although it has been shown that this species is able to survive in the wild with very low values of *N*_*e*_, the possibility of inbreeding depression in captivity should be considered, since the strategies used by this species to maintain high levels of genetic diversity in the wild despite the relatively low number of individuals are not known, or may not be possible in captivity. Genetic drift erodes genetic variation as *N*_*e*_ decreases (Allentoft & O’Brien, 2010; Montgomery et al., 2000), elevating the probability of fixation of deleterious alleles, and reducing the effectiveness of selection. This may lead to a reduction of overall fitness by limiting adaptive responses (Hare et al., 2011). Animal models suggest that, in captivity, a *N*_*e*_ > 50 is needed to avoid the risk of inbreeding depression (Frankham, Ballou & Briscoe, 2010; Traill et al., 2010). Thus, efforts to enhance the *N*_*e*_ of the captive population of *C. arnoldi* captive should be undertaken.

In keeping with the wild situation, a higher allelic richness observed in the eastern dataset when compared to the western one (Valbuena-Ureña, Amat & Carranza, 2013; Valbuena-Ureña et al., 2017). Further, the high levels of genetic differentiation observed between the eastern and western wild and captive populations suggests that the maintenance of two separated breeding lines is needed. Although outcrossing the two management units may help to increase the genetic diversity (Frankham, Ballou & Briscoe, 2010), we do not recommend it at this point for two main reasons: (a) the importance of maintaining the two genetically and morphologically differentiated groups; and (b) the potential threat to the existing populations due to outbreeding depression (OD) (Allendorf & Luikart, 2007). Existing conservation guidelines might focus on maintaining high levels of genetic diversity within the two distinct breeding lines. In the case that captive-bred individuals are introduced into brooks currently not occupied by this species, it is important to consider the eastern or western geographical position of the brook, in order to avoid possible future interbreeding as a result habitat reconnection following floods in the rivers of each sector.

### Q2: increasing the captive breeding population

Increasing the sample size clearly results in a better representation of alleles and expected heterozygosities between the simulated and the empirical datasets. The genetic distance between these two datasets also decreases when sample size is increased. The probability that a particular allele will be included among the founders is determined by its frequency in the population. Although the presence of rare alleles does not contribute much to the global genetic diversity of a certain species, they a enhance populations distinctiveness and adaptive potential. However, as seen in Fig. 4, a very large number of founders (>40 founders by sector/cluster) would be required to represent 80% of the rare alleles. In order to avoid extra costs associated with maintaining oversized captive stocks, it is crucial to detect the point at which increasing the sample size will not result in a significant benefit in terms of allelic representation (e.g., retaining all common alleles and the maximum number of rare alleles, or maximizing the mean expected heterozygosity of the founders; Lacy, 1994). This sample size is defined as the minimum captive stock size to meet the genetic goals.

Our results suggest that a minimum of 20–25 individuals for each captive breeding line should be kept in order to maintain good representation of the genetic diversity. Our results are in accordance with the Amphibian Ark guidelines (Schad, 2008) and Lacy (1994), which recommend breeding at least 20 unrelated founders by sector, with an equal sex ratio.

The expected heterozygosity (*H*_*E*_) of the eastern captive stock is significantly lower than that of the wild populations (*p* = 0.0082), indicating that the eastern captive stock only partially represents the gene pool of the eastern wild populations. Captive individuals of this breeding line originated from a single population (A1). As suggested by previous analyses of both mitochondrial and nuclear data, the eastern populations are grouped into two clusters, population A3 being the most differentiated and genetically diverse, and populations A1 and A2 clustering together in the microsatellite analysis (Valbuena-Ureña et al., 2017). Therefore, we recommended to introduce further individuals captured mainly from population A3.

In the western breeding line, in order to reduce the relatedness detected (Fig. 3), and to avoid the possibility of inbreeding, an increase of breeding individuals from this sector is also recommended. According to microsatellite loci data, the western sector clusters into two groups, the main one formed by populations B1, B2 and B4, and the other by population B3, which is separated into a distinct cluster (Valbuena-Ureña et al., 2017). Whereas the overall *H*_*E*_ of the western wild populations is 0.352, the heterozygosity increases up to 0.423 when population B3 is excluded. The low genetic diversity (*H*_*E*_ = 0.197) detected in this latter population biases the mean *H*_*E*_ value of the western wild populations. Currently, the western captive line comprises no founders from B3, but as a result of its low genetic diversity, it is unadvisable to include breeders from this site. Therefore, if additional founders are incorporated into this breeding line, individuals from B1, B2 and B4 should be prioritized.

### A need for periodic re-evaluation

Similar values of rarefied allelic richness are found among founders and descendants, which indicates that the descendants inherited almost all the microsatellite alleles from the founders. Eleven and two new PAs were found in the descendant group of the eastern and western sectors respectively; these were not detected in the founders. This could be explained by the fact that some founder females were already gravid when they were caught in the wild, so the genotype of the males was unknown. Also, some females may have fertilized their eggs with sperm from wild males stored in their reproductive apparatus, using a strategy suspected within *C. arnoldi* (Alonso, 2013) and present in many urodel species (Kühnel, Reinhard & Kupfer, 2010). Therefore, it is highly recommended that the F2, F3, etc. descendants are genotyped in order to ensure that genetic diversity is maintained over time.

Although no signs of a recent bottleneck are detected by BOTTLENECK (more effective at detecting very recent bottlenecks of low magnitude), *M*-ratio results (best suited for detecting more severe and older bottlenecks) suggested that a past bottleneck occurred in both breeding lines. Thus, it is crucial to keep monitoring for population bottlenecks in managed species since a reduction in *N*_*e*_ may enhance the rate of inbreeding, loss of genetic variation, and fixation of deleterious alleles considerably; thereby increasing the risk of population extinction (Luikart et al., 1998). As the captive breeding program of the Montseny brook newt was established very recently (less than 10 years ago) it is essential that signs of reduced genetic variation or a founder effect are monitored. Therefore, the captive stock and subsequent cohorts should be scrutinised over the long-term in order to effectively preserve genetic variation.

The current breeding protocol widely used by many captive breeding programs to minimize loss of genetic diversity involves pairing individuals according to the mean kinship value (MK) (Willoughby et al., 2015), which is based on the number and degree of relatedness of relatives that an individual has within the captive stock. The use of molecular data has proved useful to investigate the relatedness among individuals, to allow for the design of optimal mating groups, as well as allowing the identification of the natural population from which the founders originated. This underlines the importance of using molecular markers to evaluate genetic management of captive breeding programs. Therefore, further analyses on the resolution of the pedigree are needed to avoid possible mating of closely related individuals, allow deterministic parental assignment of offspring, and design optimal mating groups for maximizing diversity and avoiding genetic erosion in captivity.

Finally, a further problem associated with small founder populations is their significant rapid adaptation to captivity (Araki, Cooper & Blouin, 2007; Frankham, 2008; Gilligan & Frankham, 2003; Griffiths & Pavajeau, 2008; Witzenberger & Hochkirch, 2011). A possible solution would be to occasionally renew the captive populations with previously genotyped individuals from the wild, thereby reducing adaptation to captivity to a minimum (Williams & Hoffman, 2009; Montgomery et al., 2010).

##  Supplemental Information

10.7717/peerj.3447/supp-1 Table S1 Microsatellite allele frequencies in the wild and captive populations of the Montseny brook newt (*Calotriton arnoldi*) Sample sizes at each locus are also provided. Data from wild specimens were obtained from Valbuena-Ureña et al. (2017).

10.7717/peerj.3447/supp-2 Table S2 Estimates of genetic parameters for each breeding line and locus N, sample size; A, number of alleles per locus; Ar, allelic richness; PA, number of private alleles; *H*_*O*_, observed heterozygosity; *H*_*E*_, expected heterozygosity; *F*_IS_, inbreeding coefficient. Values in bold indicate statistical significance after Bonferroni correction.

10.7717/peerj.3447/supp-3 Table S3 Rate of missclassification for the related for the relatedness indices of Queller and Goodnight and Lynch and Ritland Rate of misclassification of unrelated (un) to half-siblings (hs) and first-order relatives (fs, full-siblings and po, parent-offsprings) for the related for the relatedness indices of Queller & Goodnight (1989, *r*_qg89_) and Lynch & Ritland (1999, *r*_lr99_) based on the midpoint between the distributions of relatedness values calculated from 10,000 simulated dyads of each relatedness category using the population allele frequencies estimated for each sample, as described in Blouin et al. (1996).

10.7717/peerj.3447/supp-4 Table S4 Cut-off values (midpoints between the means of the distributions of pairwise relatedness estimates; Blouin et al., 1996) of each simulated relationship category Un, unrelated; hs, half-sibling; fs, full-siblings; po, parent-offsprings; rel, related, including hs, fs and po. The relationship category compatible with the observed *r*_lr99_ value was and then determined for each individual-pair.

10.7717/peerj.3447/supp-5 Table S5 ANOVA and Tukey HSD posthoc comparisons among the simulated (mean of the 100 random replicates per each sample size) and empirical estimation ANOVA and Tukey HSD posthoc comparisons among the simulated (mean of the 100 random replicates per each sample size) and empirical estimations of mean percentage of common alleles (frequency >0.05), mean expected heterozygosity (*H*_*E*_) and the mean pairwise *F*_ST_ by each captive dataset (eastern sector, western sector, cluster A1A2 and cluster B1B2B4).

10.7717/peerj.3447/supp-6 Table S6 Raw data

## Additional Information and Declarations

### DNA Deposition

The following information was supplied regarding the deposition of DNA sequences: The microsatelli loci data was supplied as a Supplementary File.

### Data Availability

The following information was supplied regarding data availability: The raw data was supplied as a Supplementary File.

## References

[ref-1] Allendorf FW, Luikart G (2007). Conservation and the genetics of populations.

[ref-2] Allentoft M, O’Brien J (2010). Global amphibian declines, loss of genetic diversity and fitness: a review. Diversity.

[ref-3] Alonso M (2013). Influència de l’edat en la cria en captivitat del Tritó del Montseny (*Calotriton arnoldi*). Treball final de carrera.

[ref-4] Amat F, Carranza S (2005). Estudi demogràfic del tritó del Montseny *(Calotriton arnoldi)* al Parc Natural i Reserva de la Biosfera del Montseny.

[ref-5] Amat F, Carranza S, Valbuena-Ureña E, Carbonell F (2014). Saving the Montseny brook newt (*Calotriton arnoldi*) from extinction: an assessment of eight years of research and conservation. FroLog.

[ref-6] Amos W, Balmford A (2001). When does conservation genetics matter?. Heredity.

[ref-7] Araki H, Cooper B, Blouin MS (2007). Genetic effects of captive breeding cause a rapid, cumulative fitness decline in the wild. Science.

[ref-8] Ballou JD, Foose TJ, Kleiman D, Lumpkin S, Allen M, Harris H, Thompson K (1996). Demographic and genetic management of captive populations. Wild mammals in captivity.

[ref-9] Ballou JD, Lacy RC, Ballou JD, Gilpin E, Foose TJ (1995). Identifying genetically important individuals for management of genetic variation in pedigreed populations. Population management for survival and recovery: analytical methods and strategies in small population conservation.

[ref-10] Ballou JD, Lees C, Faust LJ, Long S, Lynch C, Lackey LB, Foose TJ, Kleiman D, Thompson KV, Bear CK (2010). Demographic and genetic management of captive populations. Wild mammals in captivity: principles and techniques for zoo management.

[ref-11] Ballou JD, Miller PS, Xie Z, Wei R, Zhang H, Zhang A, Huang S, Sun S, David VA, O’Brien SJ, Traylor-Holzer K, Wildt DE, Zhang A, Zhang H, Donal L, Janssen D, S Ellis (2006). Analysis of demographic and genetic trends for developing a captive breeding masterplan for the giant panda. Giant pandas: biology, veterinary medicine and management.

[ref-12] Blouin MS, Parsons M, Lacaille V, Lotz S (1996). Use of microsatellite loci to classify individuals by relatedness. Molecular Ecology.

[ref-13] Briscoe DA, Malpica JM, Robertson A, Smith GJ, Frankham R, Banks RG, Barker JSF (1992). Rapid loss of genetic variation in large captive populations of *Drosophila* flies: implications for the genetic management of captive populations. Conservation Biology.

[ref-14] Carbonell F, Obon E, Alonso M, Valbuena-Ureña E, Larios R, Such-Sanz Mayné J, Pomarol M, Á, Mayné J, Pomarol M (2016). Reserva genètica i cria en captivitat del tritó del Montseny (*Calotriton arnoldi*). Activitats realitzades pel centre de fauna de Torreferrussa Informe inèdit.

[ref-15] Carranza S, Amat F (2005). Taxonomy, biogeography and evolution of *Euproctus* (Amphibia: Salamandridae), with the resurrection of the genus *Calotriton* and the description of a new endemic species from the Iberian Peninsula. Zoological Journal of the Linnean Society.

[ref-16] Carranza S, Martínez-Solano I (2009). www.iucnredlist.org.

[ref-17] Conde DA, Flesness N, Colchero F, Jones OR, Scheuerlein A (2011). Zoos can lead the way with *ex situ* conservation. WAZA Magazine.

[ref-18] Dolman PM, Collar NJ, Scotland KM, Burnside RJ (2015). Ark or park: the need to predict relative effectiveness of *ex situ* and *in situ* conservation before attempting captive breeding. Journal of Applied Ecology.

[ref-19] Drechsler A, Geller D, Freund K, Schmeller DS, Künzel S, Rupp O, Loyau A, Denoël M, Valbuena-Ureña E, Steinfartz S (2013). What remains from a 454 run: estimation of success rates of microsatellite loci development in selected newt species (*Calotriton asper*, *Lissotriton helveticus*, and *Triturus cristatus*) and comparison with Illumina-based approaches. Ecology and Evolution.

[ref-20] Edwards T, Cox EC, Buzzard V, Wiese C, Hillard LS, Murphy RW (2014). Genetic assessments and parentage analysis of captive bolson tortoises (*Gopherus flavomarginatus*) inform their “rewilding” in New Mexico. PLOS ONE.

[ref-21] Excoffier L, Lischer HEL (2010). Arlequin suite ver 3.5: a new series of programs to perform population genetics analyses under Linux and Windows. Molecular Ecology Resources.

[ref-22] Frankham R (1995). Conservation genetics. Annual Review of Genetics.

[ref-23] Frankham R (2003). Genetics and conservation biology. Comptes Rendus Biologies.

[ref-24] Frankham R (2008). Genetic adaptation to captivity in species conservation programs. Molecular Ecology.

[ref-25] Frankham R, Ballou JD, Briscoe DA (2010). Introduction to conservation genetics.

[ref-26] Garza JC, Williamson EG (2001). Detection of reduction in population size using data from microsatellite loci. Molecular Ecology.

[ref-27] Gilligan DM, Frankham R (2003). Dynamics of genetic adaptation to captivity. Conservation Genetics.

[ref-28] Gonçalves da Silva A, Russello M (2009). iRel v1.0: an online tool for estimating relatedness.

[ref-29] Goudet J (1995). FSTAT (Version 1.2): a computer program to calculate F-statistics. Journal of Heredity.

[ref-30] Government of Catalonia (2010). http://mediambient.gencat.cat/.

[ref-31] Griffiths RA, Pavajeau L (2008). Captive breeding, reintroduction, and the conservation of amphibians. Conservation Biology.

[ref-32] Hale ML, Burg TM, Steeves TE (2012). Sampling for microsatellite-based population genetic studies: 25 to 30 individuals per population is enough to accurately estimate allele frequencies. PLOS ONE.

[ref-33] Harding G, Griffiths RA, Pavajeau L (2015). Developments in amphibian captive breeding and reintroduction programs. Conservation Biology.

[ref-34] Hare MP, Nunney L, Schwartz MK, Ruzzante DE, Burford M, Waples RS, Ruegg K, Palstra F (2011). Understanding and estimating effective population size for practical application in marine species management. Conservation Biology.

[ref-35] Hedrick PW, Kalinowski ST (2000). Inbreeding depresion in conservation biology. Annual Review of Ecology and Systematics.

[ref-36] Hoffmann M, Hilton-Taylor C, Angulo A, Böhm M, Brooks TM, Butchart SHM, Carpenter KE, Chanson J, Collen B, Cox NA, Darwall WRT, Dulvy NK, Harrison LR, Katariya V, Pollock CM, Quader S, Richman NI, Rodrigues ASL, Tognelli MF, Vié J-C, Aguiar JM, Allen DJ, Allen GR, Amori G, Ananjeva NB, Andreone F, Andrew P, Ortiz ALA, Baillie JEM, Baldi R, Bell BD, Biju SD, Bird JP, Black-Decima P, Blanc JJ, Bolaños F, Bolivar GW, Burfield IJ, Burton JA, Capper DR, Castro F, Catullo G, Cavanagh RD, Channing A, Chao NL, Chenery AM, Chiozza F, Clausnitzer V, Collar NJ, Collett LC, Collette BB, Fernandez CFC, Craig MT, Crosby MJ, Cumberlidge N, Cuttelod A, Derocher AE, Diesmos AC, Donaldson JS, Duckworth JW, Dutson G, Dutta SK, Emslie RH, Farjon A, Fowler S, Freyhof J, Garshelis DL, Gerlach J, Gower DJ, Grant TD, Hammerson GA, Harris RB, Heaney LR, Hedges SB, Hero J-M, Hughes B, Hussain SA, Icochea MJ, Inger RF, Ishii N, Iskandar DT, Jenkins RKB, Kaneko Y, Kottelat M, Kovacs KM, Kuzmin SL, La Marca E, Lamoreux JF, Lau MWN, Lavilla EO, Leus K, Lewison RL, Lichtenstein G, Livingstone SR, Lukoschek V, Mallon DP, McGowan PJK, McIvor A, Moehlman PD, Molur S, Alonso AM, Musick JA, Nowell K, Nussbaum RA, Olech W, Orlov NL, Papenfuss TJ, Parra-Olea G, Perrin WF, Polidoro BA, Pourkazemi M, Racey PA, Ragle JS, Ram M, Rathbun G, Reynolds RP, Rhodin AGJ, Richards SJ, Rodríguez LO, Ron SR, Rondinini C, Rylands AB, Sadovy de Mitcheson Y, Sanciangco JC, Sanders KL, Santos-Barrera G, Schipper J, Self-Sullivan C, Shi Y, Shoemaker A, Short FT, Sillero-Zubiri C, Silvano DL, Smith KG, Smith AT, Snoeks J, Stattersfield AJ, Symes AJ, Taber AB, Talukdar BK, Temple HJ, Timmins R, Tobias JA, Tsytsulina K, Tweddle D, Ubeda C, Valenti SV, Paul van Dijk P, Veiga LM, Veloso A, Wege DC, Wilkinson M, Williamson EA, Xie F, Young BE, Akçakaya HR, Bennun L, Blackburn TM, Boitani L, Dublin HT, Da Fonseca GAB, Gascon C, Lacher TE, Mace GM, Mainka SA, McNeely JA, Mittermeier RA, Reid GM, Rodriguez JP, Rosenberg AA, Samways MJ, Smart J, Stein BA, Stuart SN (2010). The impact of conservation on the status of the world’s vertebrates. Science.

[ref-37] Höglund J (2009). Evolutionary conservation genetics.

[ref-38] IUCN/SSC (2014). https://portals.iucn.org/library/sites/library/files/documents/2014-064.pdf.

[ref-39] Kalinowski ST (2005). Hp-rare 1.0: a computer program for performing rarefaction on measures of allelic richness. Molecular Ecology Notes.

[ref-40] Kühnel S, Reinhard S, Kupfer A (2010). Evolutionary reproductive morphology of amphibians: an overview. Bonn Zoological Bulletin.

[ref-41] Lacy RC, Bowles ML, Whelan CJ (1994). Managing genetic diversity in captive populations of animals. Restoration of endangered species.

[ref-42] Lacy RC (1997). Importance of genetic variation to the viability of mammalian populations. Journal of Mammalogy.

[ref-43] Leus K, Traylor-Holzer K, Lacy RC (2011). Genetic and demographic population management in zoos and aquariums: recent developments, future challenges and opportunities for scientific research. International Zoo Yearbook.

[ref-44] Lewis PO, Zaykin D (2000).

[ref-45] Luikart G, Allendorf F, Cornuet JM, Sherwin W (1998). Distortion of allele frequency distributions provides a test for recent population bottlenecks. Journal of Heredity.

[ref-46] Luikart G, Cornuet JM (1998). Empirical evaluation of a test for identifying recently bottlenecked populations from allele frequency data. Conservation Biology.

[ref-47] Lyles AM, May RM (1987). Problems in leaving the ark. Nature.

[ref-48] Lynch M, Ritland K (1999). Estimation of pairwise relatedness with molecular markers. Genetics.

[ref-49] Mace G (1986). Genetic management of small populations. International Zoo Yearbook.

[ref-50] Mace G, Pemberton JM, Stanley H (1992). Conserving genetic diversity with the help of biotechnology-desert antelopes as an example. Biotechnology and the conservation of genetic diversity Proc symposium, London.

[ref-51] Martel A, Blooi M, Adriaensen C, Van Rooij P, Beukema W, Fisher MC, Farrer RA, Schmidt BR, Tobler U, Goka K, Lips KR, Muletz C, Zamudio KR, Bosch J, Lötters S, Wombwell E, Garner TWJ, Cunningham AA, Spitzen-van der Sluijs A, Salvidio S, Ducatelle R, Nishikawa K, Nguyen TT, Kolby JE, Van Bocxlaer I, Bossuyt F, Pasmans F (2014). Recent introduction of a chytrid fungus endangers Western Palearctic salamanders. Science.

[ref-52] McCleery R, Hostetler JA, Oli MK (2014). Better off in the wild? Evaluating a captive breeding and release program for the recovery of an endangered rodent. Biological Conservation.

[ref-53] McGowan PJK, Traylor-Holzer K, Leus K (2016). IUCN Guidelines for determining when and how *ex situ* management should be used in species Conservation. Conservation Letters.

[ref-54] Montgomery ME, Woodworth LM, England PR, Briscoe DA, Frankham F (2010). Widespread selective sweeps affecting microsatellites in Drosophila populations adapting to captivity: implications for captive breeding programs. Biological Conservation.

[ref-55] Montgomery M, Woodworth L, Nurthen R, Gilligan D, Briscoe D, Frankham R (2000). Relationships between population size and loss of genetic diversity: comparisons of experimental results with theoretical predictions. Conservation Genetics.

[ref-56] Obon E, Carbonell F, Valbuena-Ureña E, Alonso M, Larios R, Fernández-Beaskoetxea S, Fisher MC, Bosch J (2013). Chytridiomycosis surveillance in the critically endangered Montseny brook newt, *Calotriton arnoldi*, northeastern Spain. The Herpetological Journal.

[ref-57] Piry S, Luikart G, Cornuet JM (1999). Computer note. BOTTLENECK: a computer program for detecting recent reductions in the effective size using allele frequency data. Journal of Heredity.

[ref-58] Princée FP, Ballou JD, Gilpin M, Foose TJ (1995). Overcoming the constraints of social structure and incomplete pedigree data through low-intensity genetic management. Population management for survival and recovery: analytical methods and strategies in small population conservation.

[ref-59] Queller DC, Goodnight KF (1989). Estimating relatedness using genetic markers. Evolution.

[ref-60] Ralls K, Ballou J (1986). Captive breeding programs for populations with a small number of founders. Trends in Ecology & Evolution.

[ref-61] Ralls K, Ballou J (1992). Managing genetic diversity in captive breeding and reintroduction programs. Transactions of the North American Wildlife and Natural Resources Conference.

[ref-62] Rousset F (2008). Genepop’007: a complete re-implementation of the genepop software for Windows and Linux. Molecular Ecology Resources.

[ref-63] Russello MA, Amato G (2004). *Ex situ* population management in the absence of pedigree information. Molecular Ecology.

[ref-64] Saccheri I, Kuussaari M, Kankare M, Vikman P, Fortelius W, Hanski I (1998). Inbreeding and extinction in a butterfly metapopulation. Nature.

[ref-65] Schad K (2008). Amphibian population management guidelines. Amphibian ark amphibian population management workshop.

[ref-66] Senner JW, Soulé ME, Wilcox BA (1980). Inbreeding depression and the survival of zoo populations. Conservation biology: an evolutionary-ecological perspective.

[ref-67] Slatkin M (1995). A measure of population subdivision based on microsatellite allele frequencies. Genetics.

[ref-68] Snyder NFR, Derrickson SR, Beissinger SR, Wiley JW, Smith TB, Toone WD, Miller B (1996). Limitations of captive breeding in endangered species recovery. Conservation Biology.

[ref-69] Soulé M, Gilpin M, Conway W, Foose T (1986). The millenium ark: how long a voyage, how many staterooms, how many passengers?. Zoo Biology.

[ref-70] Spielman D, Brook BW, Frankham R (2004). Most species are not driven to extinction before genetic factors impact them. Proceedings of the National Academy of Sciences of the United States of America.

[ref-71] Spitzen-van der Sluijs A, Martel A, Asselberghs J, Bales EK, Beukema W, Bletz MC, Dalbeck L, Goverse E, Kerres A, Kinet T, Kirst K, Laudelout A, Marin da Fonte LF, Nöllert A, Ohlhoff D, Sabino-Pinto J, Schmidt BR, Speybroeck J, Spikmans F, Steinfartz S, Veith M, Vences M, Wagner N, Pasmans F, Lötters S (2016). Expanding distribution of lethal amphibian fungus *Batrachochytrium salamandrivorans* in Europe. Emerging Infectious Diseases.

[ref-72] Tallmon DA, Koyuk A, Luikart G, Beaumont MA (2008). ONeSAMP: a program to estimate effective population size using approximate Bayesian computation. Molecular Ecology Resources.

[ref-73] Traill LW, Brook BW, Frankham RR, Bradshaw CJA (2010). Pragmatic population viability targets in a rapidly changing world. Biological Conservation.

[ref-74] Traylor-Holzer K (2011). Identifying gaps and opportunities for inter-regional *ex situ* species management. WAZA Magazine.

[ref-75] Valbuena-Ureña E, Amat F, Carranza S (2013). Integrative phylogeography of *Calotriton* newts (Amphibia, Salamandridae), with special remarks on the conservation of the endangered Montseny brook newt (*Calotriton arnoldi*). PLOS ONE.

[ref-76] Valbuena-Ureña E, Soler-Membrives A, Steinfartz S, Orozco-terWengel P, Carranza S (2017). No signs of inbreeding despite long-term isolation and habitat fragmentation in the critically endangered Montseny brook newt (*Calotriton arnoldi*). Heredity.

[ref-77] Valbuena-Ureña E, Steinfartz S, Carranza S (2014). Characterization of microsatellite loci markers for the critically endangered Montseny brook newt (*Calotriton arnoldi*). Conservation Genetics Resources.

[ref-78] Van Oosterhout C, Hutchinson WF, Wills DPM, Shipley P (2004). Micro-checker: software for identifying and correcting genotyping errors in microsatellite data. Molecular Ecology Notes.

[ref-79] Wake DB, Vredenburg VT (2008). Are we in the midst of the sixth mass extinction? A view from the world of amphibians. Proceedings of the National Academy of Sciences of the United States of America.

[ref-80] Weir BS, Cockerham CC (1984). Estimating F-statistics for the analysis of population structure. Evolution.

[ref-81] Wilcoxon F (1945). Individual comparisons by ranking methods. Biometrics Bulletin.

[ref-82] Williams SE, Hoffman EA (2009). Minimizing genetic adaptation in captive breeding programs: a review. Biological Conservation.

[ref-83] Willis K (1993). Use of animals with unknown ancestries in scientifically managed breeding programs. Zoo Biology.

[ref-84] Willis K, Willis RE (2010). How many founders, how large a population?. Zoo Biology.

[ref-85] Willoughby JR, Fernandez NB, Lamb MC, Ivy JA, Lacy RC, DeWoody JA (2015). The impacts of inbreeding, drift and selection on genetic diversity in captive breeding populations. Molecular Ecology.

[ref-86] Witzenberger K, Hochkirch A (2011). *Ex situ* conservation genetics: a review of molecular studies on the genetic consequences of captive breeding programmes for endangered animal species. Biodiversity and Conservation.

