# Supplementary material for: Getting off to a good start? Genetic evaluation of the ex situ conservation project of the Critically Endangered Montseny brook newt (Calotriton arnoldi)
Source: PeerJ. 2017 Jun 13;5:e3447. doi: 10.7717/peerj.3447 (PMC5472038; doi:10.7717/peerj.3447)
Supplement: Table S1 — Sample sizes at each locus are also provided. Data from wild specimens were obtained from Valbuena-Ureña et al. (2017). [file peerj-05-3447-s001.docx]

Table S1. Microsatellite allele frequencies in the wild and captive populations of the Montseny brook newt (*Calotriton arnoldi*). Sample sizes at each locus are also provided. Data from wild specimens were obtained from Valbuena-Ureña et al. (2017).

|  |  | **Eastern** | |  | **Western** | |
| --- | --- | --- | --- | --- | --- | --- |
| **Locus** | **Allele size (bp)** | **Wild** | **Captive** |  | **Wild** | **Captive** |
| **Calarn02248** | **169** | - | - |  | 0,072 | 0,094 |
|  | **173** | - | - |  | 0,711 | 0,844 |
|  | **177** | - | - |  | 0,217 | 0,063 |
|  | **185** | 0,006 | 0,038 |  | - | - |
|  | **193** | 0,045 | 0,212 |  | - | - |
|  | **197** | 0,357 | 0,192 |  | - | - |
|  | **201** | 0,487 | 0,404 |  | - | - |
|  | **205** | 0,097 | 0,115 |  | - | - |
|  | **209** | 0,006 | 0,038 |  | - | - |
| **Ca3** | **233** | - | - |  | 0,096 | 0,125 |
|  | **237** | - | - |  | 0,681 | 0,375 |
|  | **241** | - | - |  | 0,066 | 0,188 |
|  | **245** | - | - |  | 0,157 | 0,313 |
|  | **249** | 0,201 | 0,038 |  | - | - |
|  | **253** | 0,435 | 0,731 |  | - | - |
|  | **257** | 0,273 | 0,231 |  | - | - |
|  | **261** | 0,084 | - |  | - | - |
|  | **265** | 0,006 | - |  | - | - |
| **Calarn29994** | **169** | 0,026 | 0,058 |  | - | - |
|  | **173** | 0,961 | 0,942 |  | 0,018 | - |
|  | **177** | 0,013 | - |  | 0,735 | 0,500 |
|  | **181** | - | - |  | 0,199 | 0,406 |
|  | **185** | - | - |  | 0,048 | 0,094 |
| **Ca21** | **232** | - | - |  | 0,572 | 0,531 |
|  | **236** | 0,117 | - |  | 0,410 | 0,438 |
|  | **240** | 0,266 | 0,423 |  | 0,012 | 0,031 |
|  | **244** | 0,487 | 0,577 |  | - | - |
|  | **248** | 0,097 | - |  | - | - |
|  | **252** | 0,032 | - |  | - | - |
|  | **257** | - | - |  | 0,006 | - |
| **Calarn37825** | **211** | - | - |  | 0,030 | 0,094 |
|  | **215** | - | - |  | 0,229 | 0,250 |
|  | **219** | - | - |  | 0,036 | - |
|  | **223** | - | - |  | 0,012 | 0,031 |
|  | **227** | 0,019 | - |  | 0,006 | - |
|  | **231** | 0,071 | 0,038 |  | 0,157 | 0,125 |
|  | **235** | 0,149 | 0,058 |  | 0,392 | 0,188 |
|  | **239** | 0,149 | 0,038 |  | 0,127 | 0,313 |
|  | **243** | 0,240 | 0,019 |  | - | - |
|  | **247** | 0,208 | 0,462 |  | 0,012 | - |
|  | **251** | 0,149 | 0,346 |  | - | - |
|  | **255** | 0,013 | 0,038 |  | - | - |
| **Calarn14961** | **201** | 0,416 | 0,212 |  | - | - |
|  | **205** | 0,084 | - |  | 0,012 | - |
|  | **209** | 0,045 | 0,135 |  | 0,386 | 0,438 |
|  | **213** | 0,266 | 0,615 |  | 0,560 | 0,469 |
|  | **217** | 0,039 | - |  | 0,018 | 0,094 |
|  | **221** | 0,130 | 0,038 |  | 0,006 | - |
|  | **225** | 0,019 | - |  | 0,018 | - |
| **Calarn15906** | **105** | - | - |  | 0,018 | - |
|  | **108** | 0,006 | - |  | 0,934 | 0,969 |
|  | **113** | 0,156 | 0,442 |  | 0,048 | 0,031 |
|  | **116** | 0,481 | 0,173 |  | - | - |
|  | **121** | 0,221 | 0,308 |  | - | - |
|  | **125** | 0,110 | 0,077 |  | - | - |
|  | **129** | 0,026 | - |  | - | - |
| **Calarn12022** | **220** | 0,019 | - |  | - | - |
|  | **224** | 0,039 | 0,077 |  | 0,024 | 0,094 |
|  | **226** | 0,506 | 0,519 |  | - | - |
|  | **232** | 0,065 | 0,192 |  | 0,006 | - |
|  | **236** | 0,058 | 0,077 |  | 0,337 | 0,313 |
|  | **240** | 0,091 | - |  | 0,470 | 0,531 |
|  | **244** | 0,136 | - |  | 0,163 | 0,063 |
|  | **248** | 0,052 | 0,135 |  | - | - |
|  | **252** | 0,032 | - |  | - | - |
| **Calarn06881** | **154** | 0,026 | - |  | - | - |
|  | **158** | 0,097 | - |  | - | - |
|  | **162** | 0,175 | 0,135 |  | - | - |
|  | **166** | 0,571 | 0,750 |  | - | - |
|  | **170** | 0,019 | - |  | 0,006 | - |
|  | **174** | 0,110 | 0,115 |  | 0,108 | 0,188 |
|  | **178** | - | - |  | 0,181 | 0,188 |
|  | **182** | - | - |  | 0,639 | 0,594 |
|  | **186** | - | - |  | 0,060 | 0,031 |
|  | **190** | - | - |  | 0,006 | - |
| **Ca7** | **224** | 0,305 | 0,250 |  | - | - |
|  | **228** | 0,532 | 0,288 |  | - | - |
|  | **232** | 0,117 | 0,327 |  | 0,241 | 0,031 |
|  | **236** | 0,045 | 0,135 |  | 0,133 | 0,344 |
|  | **240** | - | - |  | 0,608 | 0,625 |
|  | **244** | - | - |  | 0,018 | - |
| **Ca22** | **126** | - | - |  | 0,500 | 0,625 |
|  | **130** | 0,006 | - |  | - | - |
|  | **134** | 0,753 | 0,981 |  | - | - |
|  | **138** | 0,227 | 0,019 |  | 0,414 | 0,344 |
|  | **142** | 0,013 | - |  | 0,043 | - |
|  | **146** | - | - |  | 0,043 | 0,031 |
| **Calarn50748** | **201** | 0,013 | - |  | - | - |
|  | **205** | 0,117 | 0,038 |  | - | - |
|  | **209** | 0,675 | 0,519 |  | 0,319 | 0,063 |
|  | **213** | 0,169 | 0,423 |  | 0,295 | 0,500 |
|  | **217** | 0,026 | 0,019 |  | 0,386 | 0,438 |
| **CA8** | **180** | - | - |  | 0,319 | 0,125 |
|  | **184** | 0,026 | 0,019 |  | 0,651 | 0,813 |
|  | **188** | 0,175 | 0,019 |  | 0,030 | 0,063 |
|  | **192** | 0,506 | 0,596 |  | - | - |
|  | **196** | 0,110 | 0,250 |  | - | - |
|  | **200** | 0,091 | 0,096 |  | - | - |
|  | **204** | 0,091 | - |  | - | - |
|  | **208** | - | 0,019 |  | - | - |
| **Calarn36791** | **120** | 0,110 | - |  | - | - |
|  | **124** | 0,013 | - |  | 0,036 | 0,219 |
|  | **128** | 0,058 | 0,058 |  | 0,861 | 0,594 |
|  | **132** | 0,006 | - |  | 0,102 | 0,188 |
|  | **136** | 0,201 | 0,519 |  | - | - |
|  | **140** | 0,422 | 0,192 |  | - | - |
|  | **144** | 0,156 | 0,192 |  | - | - |
|  | **148** | 0,032 | 0,038 |  | - | - |
| **Calarn59202** | **212** | - | - |  | 0,169 | 0,219 |
|  | **216** | - | - |  | 0,705 | 0,531 |
|  | **220** | 0,019 | - |  | - | 0,063 |
|  | **224** | 0,688 | 0,904 |  | - | - |
|  | **228** | 0,201 | 0,058 |  | 0,120 | 0,188 |
|  | **232** | 0,065 | 0,019 |  | 0,006 | - |
|  | **236** | 0,026 | - |  | - | - |
|  | **240** | - | 0,019 |  | - | - |
| **Ca32** | **185** | 0,175 | 0,173 |  | - | - |
|  | **189** | 0,636 | 0,673 |  | - | - |
|  | **193** | 0,175 | 0,135 |  | 0,910 | 0,938 |
|  | **197** | 0,013 | 0,019 |  | 0,090 | 0,063 |
| **Calarn52354** | **220** | 0,071 | 0,019 |  | - | - |
|  | **224** | 0,643 | 0,731 |  | 0,241 | 0,281 |
|  | **228** | 0,149 | 0,115 |  | 0,235 | 0,500 |
|  | **232** | 0,019 | - |  | 0,524 | 0,219 |
|  | **236** | 0,019 | - |  | - | - |
|  | **240** | 0,097 | 0,135 |  | - | - |
| **Calarn30143** | **186** | 0,097 | - |  | - | - |
|  | **190** | 0,084 | 0,135 |  | - | - |
|  | **198** | 0,396 | 0,365 |  | - | - |
|  | **202** | 0,032 | - |  | - | - |
|  | **222** | 0,136 | - |  | 0,036 | 0,063 |
|  | **226** | 0,240 | 0,442 |  | 0,157 | 0,031 |
|  | **230** | 0,013 | 0,058 |  | 0,699 | 0,906 |
|  | **234** | - | - |  | 0,084 | - |
|  | **238** | - | - |  | 0,024 | - |
| **Calarn31321** | **148** | - | - |  | 0,066 | 0,063 |
|  | **152** | - | - |  | 0,873 | 0,813 |
|  | **157** | - | - |  | 0,042 | 0,063 |
|  | **161** | - | - |  | 0,018 | 0,063 |
|  | **173** | 0,110 | 0,038 |  | - | - |
|  | **177** | 0,052 | 0,077 |  | - | - |
|  | **181** | 0,442 | 0,481 |  | - | - |
|  | **185** | 0,156 | 0,269 |  | - | - |
|  | **189** | 0,182 | 0,115 |  | - | - |
|  | **193** | 0,058 | 0,019 |  | - | - |
| **Calarn15136** | **165** | 0,032 | 0,096 |  | 0,428 | 0,156 |
|  | **169** | 0,812 | 0,442 |  | 0,024 | - |
|  | **173** | 0,123 | 0,462 |  | 0,512 | 0,781 |
|  | **177** | 0,032 | - |  | 0,036 | 0,063 |
| **Calarn37884** | **240** | - | - |  | 0,036 | 0,031 |
|  | **244** | - | - |  | 0,783 | 0,969 |
|  | **248** | 0,279 | 0,346 |  | 0,006 | - |
|  | **252** | 0,143 | 0,019 |  | - | - |
|  | **256** | 0,305 | 0,115 |  | - | - |
|  | **260** | 0,188 | 0,519 |  | - | - |
|  | **267** | 0,084 | - |  | 0,175 | - |
| **US2** | **219** | - | - |  | 0,054 | 0,063 |
|  | **223** | 0,045 | 0,077 |  | 0,024 | - |
|  | **227** | 0,292 | 0,519 |  | 0,620 | 0,500 |
|  | **231** | 0,377 | 0,308 |  | 0,133 | 0,313 |
|  | **235** | 0,188 | 0,058 |  | 0,163 | 0,125 |
|  | **239** | 0,097 | 0,038 |  | 0,006 | - |
| **Us3** | **143** | - | - |  | 1,000 | 1,000 |
|  | **155** | 0,104 | 0,077 |  | - | - |
|  | **159** | 0,662 | 0,596 |  | - | - |
|  | **163** | 0,208 | 0,231 |  | - | - |
|  | **167** | 0,026 | 0,096 |  | - | - |
| **Us7** | **222** | 0,006 | - |  | - | - |
|  | **226** | 0,071 | 0,231 |  | - | - |
|  | **234** | 0,792 | 0,750 |  | - | - |
|  | **238** | 0,130 | - |  | - | - |
|  | **246** | - | 0,019 |  | 1,000 | 1,000 |
