# Supplementary material for: Getting off to a good start? Genetic evaluation of the ex situ conservation project of the Critically Endangered Montseny brook newt (Calotriton arnoldi)
Source: PeerJ. 2017 Jun 13;5:e3447. doi: 10.7717/peerj.3447 (PMC5472038; doi:10.7717/peerj.3447)
Supplement: Table S2 — N, sample size; A, number of alleles per locus; Ar, allelic richness; PA, number of private alleles; HO, observed heterozygosity; HE, expected heterozygosity; FIS, inbreeding coefficient. Values in bold indicate statistical significance after Bonferroni correction. [file peerj-05-3447-s002.docx]

Table S2. Estimates of genetic parameters for each breeding line and locus. N, sample size; A, number of alleles per locus; Ar, allelic richness; PA, number of private alleles; H_O_, observed heterozygosity; H_E_, expected heterozygosity; *F*_IS_, inbreeding coefficient. Values in bold indicate statistical significance after Bonferroni correction.

|  | Locus | Calarn 02248 | Ca3 | Calarn 29994 | Ca21 | Calarn 37825 | Calarn 14961 | Calarn 15906 | Calarn 12022 | Calarn 06881 | Ca7 | Ca22 | Calarn 50748 | CA8 | Calarn 36791 | Calarn 59202 | Ca 32 | Calarn 52354 | Calarn 30143 | Calarn 31321 | Calarn 15136 | Calarn 37884 | US2 | Us3 | Us7 | Overall |
| --- | --- | --- | --- | --- | --- | --- | --- | --- | --- | --- | --- | --- | --- | --- | --- | --- | --- | --- | --- | --- | --- | --- | --- | --- | --- | --- |
| Eastern founders | N | 9 | 9 | 9 | 9 | 9 | 9 | 9 | 9 | 9 | 9 | 9 | 9 | 9 | 9 | 9 | 9 | 9 | 9 | 9 | 9 | 9 | 9 | 9 | 9 | 9 |
|  | A | 5,000 | 2,000 | 2,000 | 2,000 | 5,000 | 4,000 | 4,000 | 4,000 | 3,000 | 4,000 | 1,000 | 2,000 | 4,000 | 4,000 | 3,000 | 4,000 | 2,000 | 4,000 | 4,000 | 3,000 | 4,000 | 5,000 | 4,000 | 3,000 | 3,417 |
|  | Ar | 4,617 | 1,976 | 1,902 | 2,000 | 4,471 | 3,799 | 3,662 | 3,333 | 2,642 | 3,897 | 1,000 | 2,000 | 3,471 | 3,892 | 2,333 | 3,564 | 1,902 | 3,642 | 3,569 | 2,666 | 3,642 | 4,000 | 3,657 | 2,662 | 3,100 |
|  | PA | 5 | 2 | 1 | 1 | 1 | 1 | 3 | 2 | 2 | 2 | 1 | 0 | 3 | 3 | 1 | 2 | 1 | 2 | 4 | 0 | 3 | 0 | 4 | 2 | 46 |
|  | H_O_ | 1,000 | 0,333 | 0,222 | 0,556 | 0,889 | 0,667 | 0,889 | 0,556 | 0,444 | 0,444 | 0,000 | 0,667 | 0,444 | 0,889 | 0,222 | 0,667 | 0,222 | 0,889 | 0,667 | 0,444 | 0,778 | 0,778 | 0,778 | 0,333 | 0,574 |
|  | H_E_ | 0,753 | 0,278 | 0,198 | 0,475 | 0,710 | 0,617 | 0,685 | 0,574 | 0,364 | 0,716 | 0,000 | 0,444 | 0,451 | 0,691 | 0,204 | 0,562 | 0,198 | 0,642 | 0,623 | 0,475 | 0,660 | 0,630 | 0,648 | 0,426 | 0,501 |
|  | *F*_IS_ | -0,274 | -0,143 | -0,067 | -0,111 | -0,196 | -0,021 | -0,243 | 0,091 | -0,164 | 0,429 | - | -0,455 | 0,072 | -0,231 | -0,032 | -0,129 | -0,067 | -0,333 | -0,011 | 0,123 | -0,120 | -0,179 | -0,143 | 0,273 | -0,088 |
| Eastern F1 | N | 17 | 17 | 17 | 17 | 17 | 17 | 17 | 17 | 17 | 17 | 17 | 17 | 17 | 17 | 17 | 17 | 17 | 17 | 17 | 17 | 17 | 17 | 17 | 17 | 17 |
|  | A | 6,000 | 3,000 | 2,000 | 2,000 | 6,000 | 3,000 | 4,000 | 5,000 | 3,000 | 4,000 | 2,000 | 4,000 | 5,000 | 5,000 | 3,000 | 3,000 | 4,000 | 4,000 | 6,000 | 3,000 | 3,000 | 5,000 | 3,000 | 2,000 | 3,750 |
|  | Ar | 4,508 | 2,579 | 1,353 | 2,000 | 3,646 | 2,874 | 3,643 | 4,372 | 2,687 | 3,864 | 1,353 | 2,941 | 3,446 | 3,830 | 1,941 | 2,811 | 3,203 | 3,430 | 4,221 | 2,840 | 2,742 | 3,679 | 2,825 | 1,982 | 3,030 |
|  | PA | 6 | 3 | 1 | 1 | 3 | 1 | 3 | 2 | 2 | 2 | 1 | 1 | 4 | 4 | 2 | 2 | 2 | 2 | 6 | 0 | 2 | 0 | 3 | 2 | 55 |
|  | H_O_ | 0,882 | 0,529 | 0,059 | 0,529 | 0,647 | 0,412 | 0,706 | 0,765 | 0,412 | 0,706 | 0,059 | 0,471 | 0,353 | 0,706 | 0,176 | 0,471 | 0,353 | 0,588 | 0,824 | 0,471 | 0,647 | 0,706 | 0,588 | 0,471 | 0,522 |
|  | H_E_ | 0,697 | 0,469 | 0,057 | 0,493 | 0,619 | 0,517 | 0,663 | 0,683 | 0,420 | 0,720 | 0,057 | 0,580 | 0,606 | 0,621 | 0,164 | 0,458 | 0,528 | 0,644 | 0,687 | 0,569 | 0,555 | 0,621 | 0,512 | 0,360 | 0,513 |
|  | *F*_IS_ | -0,237 | -0,099 | 0,000 | -0,043 | -0,014 | 0,233 | -0,035 | -0,089 | 0,051 | 0,050 | 0,000 | 0,217 | 0,442 | -0,107 | -0,043 | 0,004 | 0,358 | 0,116 | -0,170 | 0,202 | -0,135 | -0,107 | -0,119 | -0,280 | 0,012 |
| Eastern total captive | N | 26 | 26 | 26 | 26 | 26 | 26 | 26 | 26 | 26 | 26 | 26 | 26 | 26 | 26 | 26 | 26 | 26 | 26 | 26 | 26 | 26 | 26 | 26 | 26 | 26 |
|  | A | 6,000 | 3,000 | 2,000 | 2,000 | 7,000 | 4,000 | 4,000 | 5,000 | 3,000 | 4,000 | 2,000 | 4,000 | 6,000 | 5,000 | 4,000 | 4,000 | 4,000 | 4,000 | 6,000 | 3,000 | 4,000 | 5,000 | 4,000 | 3,000 | 4,083 |
|  | Ar | 4,543 | 2,385 | 1,553 | 2,000 | 4,016 | 3,234 | 3,582 | 4,132 | 2,672 | 3,829 | 1,231 | 2,642 | 3,420 | 3,858 | 2,015 | 3,017 | 2,903 | 3,412 | 4,103 | 2,746 | 3,040 | 3,621 | 3,382 | 2,204 | 3,060 |
|  | PA | 6 | 3 | 1 | 1 | 3 | 1 | 3 | 2 | 2 | 2 | 1 | 1 | 4 | 4 | 2 | 2 | 2 | 2 | 6 | 0 | 3 | 0 | 4 | 2 | 57 |
|  | H_O_ | 0,923 | 0,462 | 0,115 | 0,538 | 0,731 | 0,500 | 0,769 | 0,692 | 0,423 | 0,615 | 0,038 | 0,538 | 0,385 | 0,769 | 0,192 | 0,538 | 0,308 | 0,692 | 0,769 | 0,462 | 0,692 | 0,731 | 0,654 | 0,423 | 0,540 |
|  | H_E_ | 0,739 | 0,411 | 0,109 | 0,488 | 0,659 | 0,557 | 0,674 | 0,663 | 0,406 | 0,729 | 0,038 | 0,550 | 0,572 | 0,652 | 0,179 | 0,499 | **0,434** | 0,649 | 0,675 | 0,582 | 0,597 | 0,625 | 0,576 | 0,384 | 0,519 |
|  | *F*_IS_ | -0,231 | -0,103 | -0,042 | -0,084 | -0,089 | 0,122 | -0,122 | -0,024 | -0,022 | 0,175 | 0,000 | 0,040 | 0,345 | -0,161 | -0,055 | -0,061 | 0,309 | -0,047 | -0,120 | 0,226 | -0,141 | -0,150 | -0,115 | -0,083 | -0,022 |
| Western founders | N | 10 | 10 | 10 | 10 | 10 | 10 | 10 | 10 | 10 | 10 | 10 | 10 | 10 | 10 | 10 | 10 | 10 | 10 | 10 | 10 | 10 | 10 | 10 | 10 | 10 |
|  | A | 3,000 | 4,000 | 3,000 | 3,000 | 6,000 | 3,000 | 1,000 | 4,000 | 3,000 | 3,000 | 3,000 | 3,000 | 3,000 | 3,000 | 4,000 | 2,000 | 3,000 | 3,000 | 3,000 | 3,000 | 1,000 | 4,000 | 1,000 | 1,000 | 2,917 |
|  | Ar | 2,551 | 3,848 | 2,600 | 2,600 | 5,000 | 2,600 | 1,000 | 3,803 | 2,936 | 2,596 | 2,596 | 2,853 | 2,705 | 2,985 | 3,547 | 1,853 | 2,985 | 2,200 | 2,705 | 2,838 | 1,000 | 3,803 | 1,000 | 1,000 | 2,650 |
|  | PA | 3 | 4 | 2 | 1 | 3 | 0 | 0 | 0 | 2 | 1 | 2 | 0 | 1 | 0 | 2 | 0 | 0 | 0 | 4 | 0 | 1 | 1 | 1 | 0 | 28 |
|  | H_O_ | 0,200 | 0,900 | 0,400 | 0,600 | 0,600 | 0,400 | 0,000 | 0,800 | 0,500 | 0,400 | 0,400 | 0,700 | 0,300 | 0,500 | 0,600 | 0,200 | 0,800 | 0,200 | 0,300 | 0,500 | 0,000 | 0,600 | 0,000 | 0,000 | 0,413 |
|  | H_E_ | 0,335 | 0,715 | 0,515 | 0,535 | 0,740 | 0,545 | 0,000 | 0,675 | 0,515 | 0,445 | 0,445 | 0,585 | 0,340 | 0,620 | 0,610 | 0,180 | 0,635 | 0,185 | 0,340 | 0,460 | 0,000 | 0,675 | 0,000 | 0,000 | 0,421 |
|  | *F*_IS_ | 0,446 | -0,209 | 0,273 | -0,069 | 0,239 | 0,314 | - | -0,134 | 0,082 | 0,153 | 0,153 | -0,145 | 0,169 | 0,244 | 0,069 | -0,059 | -0,210 | -0,029 | 0,169 | -0,034 | - | 0,163 | - | - | 0,072 |
| Western F1 | N | 6 | 6 | 6 | 6 | 6 | 6 | 6 | 6 | 6 | 6 | 6 | 6 | 6 | 6 | 6 | 6 | 6 | 6 | 6 | 6 | 6 | 6 | 6 | 6 | 6 |
|  | A | 2,000 | 4,000 | 3,000 | 2,000 | 5,000 | 3,000 | 2,000 | 2,000 | 4,000 | 2,000 | 2,000 | 2,000 | 2,000 | 3,000 | 4,000 | 1,000 | 3,000 | 2,000 | 2,000 | 2,000 | 2,000 | 3,000 | 1,000 | 1,000 | 2,458 |
|  | Ar | 2,000 | 4,000 | 3,000 | 2,000 | 5,000 | 3,000 | 2,000 | 2,000 | 4,000 | 2,000 | 2,000 | 2,000 | 2,000 | 3,000 | 4,000 | 1,000 | 3,000 | 2,000 | 2,000 | 2,000 | 2,000 | 3,000 | 1,000 | 1,000 | 2,460 |
|  | PA | 2 | 4 | 2 | 1 | 2 | 0 | 0 | 0 | 3 | 1 | 1 | 0 | 1 | 0 | 2 | 0 | 0 | 0 | 2 | 0 | 2 | 0 | 1 | 0 | 24 |
|  | H_O_ | 0,167 | 0,667 | 1,000 | 1,000 | 1,000 | 0,833 | 0,167 | 0,333 | 0,833 | 1,000 | 1,000 | 0,500 | 0,333 | 0,500 | 0,667 | 0,000 | 0,833 | 0,167 | 0,333 | 0,167 | 0,167 | 0,500 | 0,000 | 0,000 | 0,507 |
|  | H_E_ | 0,153 | 0,681 | 0,611 | 0,500 | 0,792 | 0,625 | 0,153 | 0,444 | 0,653 | 0,500 | 0,500 | 0,486 | 0,278 | 0,403 | 0,653 | 0,000 | 0,569 | 0,153 | 0,278 | 0,153 | 0,153 | 0,542 | 0,000 | 0,000 | 0,387 |
|  | *F*_IS_ | 0,000 | 0,111 | -0,579 | -1,000 | -0,176 | -0,250 | 0,000 | 0,333 | -0,190 | -1,000 | -1,000 | 0,063 | -0,111 | -0,154 | 0,070 | - | -0,389 | 0,000 | -0,111 | 0,000 | 0,000 | 0,167 | - | - | -0,227 |
| Western total captive | N | 16 | 16 | 16 | 16 | 16 | 16 | 16 | 16 | 16 | 16 | 16 | 16 | 16 | 16 | 16 | 16 | 16 | 16 | 16 | 16 | 16 | 16 | 16 | 16 | 16 |
|  | A | 3,000 | 4,000 | 3,000 | 3,000 | 6,000 | 3,000 | 2,000 | 4,000 | 4,000 | 3,000 | 3,000 | 3,000 | 3,000 | 3,000 | 4,000 | 2,000 | 3,000 | 3,000 | 4,000 | 3,000 | 2,000 | 4,000 | 1,000 | 1,000 | 3,083 |
|  | Ar | 2,387 | 3,819 | 2,770 | 2,375 | 4,953 | 2,770 | 1,375 | 3,384 | 3,290 | 2,374 | 2,374 | 2,617 | 2,482 | 2,934 | 3,551 | 1,617 | 2,971 | 1,992 | 2,851 | 2,540 | 1,375 | 3,479 | 1,000 | 1,000 | 2,590 |
|  | PA | 3 | 4 | 2 | 1 | 3 | 0 | 0 | 0 | 3 | 1 | 2 | 0 | 1 | 0 | 2 | 0 | 0 | 0 | 4 | 0 | 2 | 1 | 1 | 0 | 30 |
|  | H_O_ | 0,188 | 0,813 | 0,625 | 0,750 | 0,750 | 0,563 | 0,063 | 0,625 | 0,625 | 0,625 | 0,625 | 0,625 | 0,313 | 0,500 | 0,625 | 0,125 | 0,813 | 0,188 | 0,313 | 0,375 | 0,063 | 0,563 | 0,000 | 0,000 | 0,448 |
|  | H_E_ | 0,275 | 0,711 | 0,576 | 0,525 | 0,779 | 0,580 | 0,061 | 0,607 | 0,576 | 0,490 | 0,490 | 0,555 | 0,320 | 0,564 | 0,631 | 0,117 | 0,623 | 0,174 | 0,328 | 0,361 | 0,061 | 0,633 | 0,000 | 0,000 | 0,418 |
|  | *F*_IS_ | 0,348 | -0,111 | -0,053 | -0,401 | 0,070 | 0,063 | 0,000 | 0,003 | -0,053 | -0,245 | -0,245 | -0,095 | 0,057 | 0,146 | 0,042 | -0,034 | -0,275 | -0,047 | 0,080 | -0,006 | 0,000 | 0,143 | - | - | -0,039 |
