# Supplementary material for: Getting off to a good start? Genetic evaluation of the ex situ conservation project of the Critically Endangered Montseny brook newt (Calotriton arnoldi)
Source: PeerJ. 2017 Jun 13;5:e3447. doi: 10.7717/peerj.3447 (PMC5472038; doi:10.7717/peerj.3447)
Supplement: Table S3 — Rate of misclassification of unrelated (un) to half-siblings (hs) and first-order relatives (fs, full-siblings and po, parent-offsprings) for the related for the relatedness indices of Queller & Goodnight (1989, rqg89) and Lynch & Ritland (1999, rlr99) based on the midpoint between the distributions of relatedness values calculated from 10,000 simulated dyads of each relatedness category using the population allele frequencies estimated for each sample, as described in Blouin et al. (1996). [file peerj-05-3447-s003.docx]

Table S3. Rate of misclassification of unrelated (un) to half-siblings (hs) and first-order relatives (fs: full-siblings and po: parent-offsprings) for the related for the relatedness indices of Queller and Goodnight (1989, r_qg89_) and Lynch and Ritland (1999, r_lr99_) based on the midpoint between the distributions of relatedness values calculated from 10000 simulated dyads of each relatedness category using the population allele frequencies estimated for each sample, as described in Blouin et al. (1996).

|  |  | Misclassified as | | | | | | | | | |
| --- | --- | --- | --- | --- | --- | --- | --- | --- | --- | --- | --- |
|  |  | r_qg89_ | | | | | r_lr99_ | | | | |
| Simulated relationship | A1A2 |  | po | fs | hs | un |  | po | fs | hs | un |
|  |  | po | 0.4960 | 0.3572 | 0.1453 | 0.0015 | po | 0.4985 | 0.3019 | 0.1989 | 0.0007 |
|  |  | fs | 0.5029 | 0.2945 | 0.1929 | 0.0097 | fs | 0.5015 | 0.2716 | 0.2217 | 0.0052 |
|  |  | hs | 0.0478 | 0.1545 | 0.5764 | 0.2213 | hs | 0.0761 | 0.1508 | 0.6063 | 0.1668 |
|  |  | un | 0.0008 | 0.0088 | 0.2156 | 0.7748 | un | 0.0006 | 0.0043 | 0.1619 | 0.8332 |
|  | B1B2B4 |  | po | fs | hs | un |  | po | fs | hs | un |
|  |  | po | 0.4991 | 0.2966 | 0.1979 | 0.0064 | po | 0.5001 | 0.2804 | 0.2153 | 0.0042 |
|  |  | fs | 0.5007 | 0.2569 | 0.2210 | 0.0214 | fs | 0.4999 | 0.2474 | 0.2381 | 0.0146 |
|  |  | hs | 0.0820 | 0.1602 | 0.5065 | 0.2513 | hs | 0.1002 | 0.1525 | 0.5438 | 0.2035 |
|  |  | un | 0.0054 | 0.0196 | 0.2263 | 0.7487 | un | 0.0023 | 0.0094 | 0.1921 | 0.7962 |
