# Supplementary material for: Getting off to a good start? Genetic evaluation of the ex situ conservation project of the Critically Endangered Montseny brook newt (Calotriton arnoldi)
Source: PeerJ. 2017 Jun 13;5:e3447. doi: 10.7717/peerj.3447 (PMC5472038; doi:10.7717/peerj.3447)
Supplement: Table S4 — Un, unrelated; hs, half-sibling; fs, full-siblings; po, parent-offsprings; rel, related, including hs, fs and po. The relationship category compatible with the observed rlr99 value was and then determined for each individual-pair. [file peerj-05-3447-s004.docx]

Table S4. Cut-off values (midpoints between the means of the distributions of pairwise relatedness estimates; Blouin et al. 1996) of each simulated relationship category. Un: unrelated, hs: half-sibling, fs: full-siblings, po: parent-offsprings, rel: related, including hs, fs and po. The relationship category compatible with the observed r_lr99_ value was and then determined for each individual-pair.

|  |  | r_lr99_ | r_qg89_ |
| --- | --- | --- | --- |
| A1A2 | un-rel | 0.2093 | 0.2525 |
|  | un | 0.0000 | 0.0000 |
|  | hs | 0.1881 | 0.2734 |
|  | 1st order | 0.4602 | 0.4973 |
| B1B2B4 | un-rel | 0.1729 | 0.2076 |
|  | un | 0.0000 | 0.0000 |
|  | hs | 0.2295 | 0.3244 |
|  | 1st order | 0.4134 | 0.4480 |
