# Supplementary material for: Getting off to a good start? Genetic evaluation of the ex situ conservation project of the Critically Endangered Montseny brook newt (Calotriton arnoldi)
Source: PeerJ. 2017 Jun 13;5:e3447. doi: 10.7717/peerj.3447 (PMC5472038; doi:10.7717/peerj.3447)
Supplement: Table S5 — ANOVA and Tukey HSD posthoc comparisons among the simulated (mean of the 100 random replicates per each sample size) and empirical estimations of mean percentage of common alleles (frequency >0.05), mean expected heterozygosity (HE) and the mean pairwise FST by each captive dataset (eastern sector, western sector, cluster A1A2 and cluster B1B2B4). [file peerj-05-3447-s005.docx]

Table S5. ANOVA and Tukey HSD posthoc comparisons among the simulated (mean of the 100 random replicates per each sample size) and empirical estimations of mean percentage of common alleles (frequency >0.05), mean expected heterozygosity (H_E_) and the mean pairwise *F*_ST_ by each captive dataset (eastern sector, western sector, cluster A1A2 and cluster B1B2B4).

|  |  | % A>0.05 detected | | He | | Fst | |
| --- | --- | --- | --- | --- | --- | --- | --- |
|  |  | ANOVA | | | | | |
|  |  | F | *P*-level | F | P-LEVEL | F | P-LEVEL |
| Eastern | | 1125.00 | **0.0000** | 169.00 |  | 1037.93 | **0.0000** |
| Western | | 574.00 | **0.0000** | 69.70 | 0.00 | 445.86 | **0.0000** |
| A1A2 | | 1170.00 | **0.0000** | 161.00 | 0.00 | 656.50 | **0.0000** |
| B1B2B4 | | 863.00 | **0.0000** | 89.20 | 0.00 | 883.56 | **0.0000** |
|  |  | Tukey HSD posthoc comparison | | | | | |
|  |  | *P* | | *P* | | *P* | |
| Eastern | 5-10 | 0.0000 | | 0.0000 | | 0.0000 | |
|  | 10-15 | 0.0000 | | 0.0041 | | 0.0000 | |
|  | 15-20 | 0.0000 | | 0.0026 | | 0.0000 | |
|  | 20-25 | **0.1366** | | **0.9967** | | 0.0011 | |
|  | 25-30 | **0.8514** | | **0.4418** | | **0.0919** | |
|  | 30-35 | **1.0000** | | **0.9978** | | **0.9454** | |
|  | 35-40 | **1.0000** | | **1.0000** | | **0.8520** | |
|  | 40-45 | **1.0000** | | **1.0000** | | **0.9574** | |
|  | 45-50 | **1.0000** | | **1.0000** | | **0.9989** | |
| Western | 5-10 | 0.0000 | | 0.0000 | | 0.0000 | |
|  | 10-15 | 0.0000 | | 0.0032 | | 0.0000 | |
|  | 15-20 | 0.0006 | | **0.6042** | | 0.0022 | |
|  | 20-25 | **0.4706** | | **1.0000** | | **0.1420** | |
|  | 25-30 | **0.9925** | | **0.5201** | | **0.8390** | |
|  | 30-35 | **1.0000** | | **1.0000** | | **0.8981** | |
|  | 35-40 | **1.0000** | | **1.0000** | | **1.0000** | |
|  | 40-45 | **1.0000** | | **1.0000** | | **0.9850** | |
|  | 45-50 | **1.0000** | | **0.9992** | | **1.0000** | |
| A1A2 | 5-10 | 0.0000 | | 0.0000 | | 0.0000 | |
|  | 10-15 | 0.0000 | | 0.0000 | | 0.0000 | |
|  | 15-20 | 0.0000 | | **0.8261** | | 0.0000 | |
|  | 20-25 | **0.7208** | | **0.9503** | | 0.0143 | |
|  | 25-30 | **0.9969** | | **0.7628** | | **0.6823** | |
|  | 30-35 | **1.0000** | | **0.9999** | | **0.9254** | |
|  | 35-40 | **1.0000** | | **1.0000** | | **0.9710** | |
|  | 40-45 | **1.0000** | | **0.9908** | | **0.9969** | |
|  | 45-50 | **1.0000** | | **0.9980** | | **0.9986** | |
| B1B2B4 | 5-10 | 0.0000 | | 0.0000 | | 0.0000 | |
|  | 10-15 | 0.0000 | | **0.6054** | | 0.0000 | |
|  | 15-20 | 0.0000 | | 0.0130 | | 0.0000 | |
|  | 20-25 | **0.2645** | | **0.9097** | | 0.0017 | |
|  | 25-30 | **0.9848** | | **1.0000** | | **0.3486** | |
|  | 30-35 | **1.0000** | | **1.0000** | | **0.9196** | |
|  | 35-40 | **1.0000** | | **0.9994** | | **0.8520** | |
|  | 40-45 | **1.0000** | | **0.9978** | | **0.9971** | |
|  | 45-50 | **1.0000** | | **1.0000** | | **0.9964** | |
